# HPV‐related methylation‐based reclassification and risk stratification of cervical cancer

**DOI:** 10.1002/1878-0261.12709

**Published:** 2020-06-02

**Authors:** Si Yang, Ying Wu, Shuqian Wang, Peng Xu, Yujiao Deng, Meng Wang, Kang Liu, Tian Tian, Yuyao Zhu, Na Li, Linghui Zhou, Zhijun Dai, Huafeng Kang

**Affiliations:** ^1^ Department of Oncology The Second Affiliated Hospital of Xi’an Jiaotong University Xi'an China; ^2^ Department of Breast Surgery The First Affiliated Hospital College of Medicine Zhejiang University Hangzhou China; ^3^ Department of Hepatobiliary Surgery The First Affiliated Hospital of Xi'an Jiaotong University Xi'an China

**Keywords:** cervical cancer, DNA methylation, human papillomavirus, prognosis, risk stratification, subtype

## Abstract

Human papillomavirus (HPV) is a clear etiology of cervical cancer (CC). However, the associations between HPV infection and DNA methylation have not been thoroughly investigated. Additionally, it remains unknown whether HPV‐related methylation signatures can identify subtypes of CC and stratify the prognosis of CC patients. DNA methylation profiles were obtained from The Cancer Genome Atlas to identify HPV‐related methylation sites. Unsupervised clustering analysis of HPV‐related methylation sites was performed to determine the different CC subtypes. CC patients were categorized into cluster 1 (Methylation‐H), cluster 2 (Methylation‐M), and cluster 3 (Methylation‐L). Compared to Methylation‐M and Methylation‐L, Methylation‐H exhibited a significantly improved overall survival (OS). Gene set enrichment analysis (GSEA) was conducted to investigate the functions that correlated with different CC subtypes. GSEA indicated that the hallmarks of tumors, including KRAS signaling, TNFα signaling via NF‐κB, inflammatory response, epithelial–mesenchymal transition, and interferon‐gamma response, were enriched in Methylation‐M and Methylation‐L. Based on mutation and copy number variation analyses, we found that aberrant mutations, amplifications, and deletions among the MYC, Notch, PI3K‐AKT, and RTK‐RAS pathways were most frequently detected in Methylation‐H. Additionally, mutations, amplifications, and deletions within the Hippo, PI3K‐AKT, and TGF‐β pathways were presented in Methylation‐M. Genes within the cell cycle, Notch, and Hippo pathways possessed aberrant mutations, amplifications, and deletions in Methylation‐L. Moreover, the analysis of tumor microenvironments revealed that Methylation‐H was characterized by a relatively low degree of immune cell infiltration. Finally, a prognostic signature based on six HPV‐related methylation sites was developed and validated. Our study revealed that CC patients could be classified into three heterogeneous clusters based on HPV‐related methylation signatures. Additionally, we derived a prognostic signature using six HPV‐related methylation sites that stratified the OS of patients with CC into high‐ and low‐risk groups.

## Graphical abstract

Cervical cancer (CC) patients could be classified into three clusters with distinct survival based on the human papillomavirus (HPV)‐related methylation sites. Specific biological processes, pathways, and genomic alterations could be correlated with the different outcomes in the three clusters. Additionally, we derived a prognostic signature using six HPV‐related methylation sites that stratified the patients with CC into high‐ and low‐risk groups.

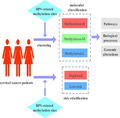

**Abbreviations**
aDCs: activated dendritic cells
AUC: the area under the curve
CC: cervical cancer
CI: confidence interval
CNV: copy number variation
DCs: dendritic cells
DMP: differentially methylated probe
ESTIMATE: Estimation of STromal and Immune cells in MAlignant Tumor tissues using Expression data
GSEA: gene set enrichment analysis
GX: unknown histological grade
HPV: human papillomavirus
HR: hazard ratio
iDCs: immature dendritic cells
LASSO: least absolute shrinkage and selection operator
NES: normalized enrichment score
NK: natural killer
NX: unknown N stage
OS: overall survival
PCA: principal component analysis
pDCs: plasmacytoid dendritic cells
ROC: receiver operating characteristic
SsGSEA: single sample gene set enrichment analysis
TCGA: The Cancer Genome Atlas
Tcm cells: central memory T cells
Tem cells: effector memory T cells
Tfh cells: follicular helper T cells
Tgd cells: gamma delta T cells
Th1 cells: type 1T helper cells
Treg cells: regulatory T cells
TSG: tumor suppressor gene
TX: unknown T stage

## 1 Introduction

Cervical cancer (CC) is a major public health concern and is the fourth most frequently diagnosed cancer and the fourth leading cause of cancer‐related death in women worldwide (Bray *et al*., 2018). A persistent infection with oncogenic human papillomavirus (HPV) can lead to cervical precancerous lesions that may ultimately develop into cancer (Crosbie *et al*., 2013). There are more than 100 identified HPV genotypes, and types 16 and 18 are the most common in CC (Munoz *et al*., 2006). HPV plays a significant role in the pathogenesis of CC; it affects apoptosis, cell cycle, cell adhesion, and DNA repair mechanisms within the host cell and can also activate immune responses (Coussens and Werb, 2002; Whiteside *et al*., 2008). The integration of HPV virus into the host genome often occurs within the transcribed genomic region, and this mechanism is utilized by the virus to increase the expression of certain viral products, including the E6 and E7 viral oncogenes (Schmitz *et al*., 2012; Ziegert *et al*., 2003). Additionally, the integration of HPV virus is strongly associated with the development of CC (Li *et al*., 2008).

Aberrant DNA methylation is an epigenetic hallmark of tumors and leads to tumor development and progression by silencing tumor suppressor genes (TSGs) and activating oncogenes (Das and Singal, 2004; Egger *et al*., 2004). The characteristics of DNA methylation make epigenetic changes ideal and clinically applicable biomarkers for diagnosis or use as prognostic indicators in cancer (Keeley *et al*., 2013). A growing number of studies have shown that aberrant DNA methylation plays a significant role in tumor progression, and DNA methylation can serve as a biomarker for predicting the prognosis of patients with a variety of tumors (Guo *et al*., 2004; Roh *et al*., 2016; Zhou *et al*., 2014). In CC, aberrant DNA methylation can occur on the integrated viral DNA, but it can also occur within the host cell genome (Yanatatsaneejit *et al*., 2011). HPV infection has been observed to be correlated with the regulation of DNA methylation in CC. Both E6 and E7 oncoproteins encoded by HPV type 16 affect the DNA methyltransferase DNMT1 (Au Yeung *et al*., 2010; Burgers *et al*., 2007). The E7 protein directly combines with DNMT1 and stimulates its DNA methyltransferase activity (Burgers *et al*., 2007). On the other hand, E6 protein has been reported to upregulate the DNMT1 through suppression of p53 (Au Yeung *et al*., 2010). The upregulation of DNA methyltransferases by HPV oncoproteins can increase methylation of the host cell genome and repress transcription of TSGs. HPV can drive progression of CC through the aberrant DNA methylation in plenty of TSGs, such as E‐cadherin (Laurson *et al*., 2010), p53 (Moody and Laimins, 2010), and RB1 (Yim and Park, 2005).

Many recent studies examining CC investigated only aberrant DNA methylation of one or a few genes using relatively small sample cohorts. These studies also ignored the association of HPV infection with DNA methylation. There are variations in the DNA methylation profiles, suggesting that novel methylation signatures are required for diagnosis and predicting outcomes of CC. Therefore, this study was conducted to determine the epigenetic alterations involved in CC progression and HPV infection. The DNA methylation profiles of CC samples from The Cancer Genome Atlas (TCGA) were analyzed. HPV‐related methylation sites were obtained in the present study. We then performed unsupervised hierarchical clustering of HPV‐related methylation sites to determine the subgroups of CC patients. The gene expression RNA‐sequencing, mutation, and copy number variation (CNV) profiles in CC patients within the different methylation subgroups were investigated. Finally, a new signature possessing predictive power based on HPV‐related methylation sites was developed and validated to stratify the prognosis of CC.

## 2 Materials and Methods

### 2.1 Data collection and processing

The Cancer Genome Atlas DNA methylation data from 309 CC samples and three adjacent samples based on the Illumina HumanMethylation 450 (450K) platform (Illumina Inc., San Diego, CA, USA) were downloaded from UCSC xena (https://xena.ucsc.edu/). The genomic annotation of the CpG sites was based on GRCh38. The methylation levels of the CpG sites were estimated as beta‐values and calculated as M/(M + U), where M is the signal from methylated beads, and U is the signal from un‐methylated beads at the targeted CpG site (Bibikova *et al*., 2011). For each CpG site, their beta‐values ranged from 0 (no DNA methylation) to 1 (100% DNA methylation) (Bibikova *et al*., 2011).

Clinical information for 307 CC patients was obtained from UCSC xena. A total of 13 CC patients were then excluded because their survival time was zero. We extracted the information regarding HPV infection status from Table S2 of the study published by TCGA (Cancer Genome Atlas Research Network, 2017).

The RNA‐sequencing gene expression, somatic mutation, and CNV profiles for 306, 289, and 297 patients with CC, respectively, were obtained from TCGA data portal (https://portal.gdc.cancer.gov/). Somatic mutation data, which stored in the form of Mutation Annotation Format, were analyzed and summarized using maftools (Mayakonda *et al*., 2018). Significant amplification or deletion alterations were determined using GISTIC 2.0 based on a robust algorithm to detect recurrent somatic CNVs by evaluating the frequency and amplitude of corresponding events (Mermel *et al*., 2011).

### 2.2 Identification and screening of HPV‐related methylation sites

ChAMP was used to perform quality control, standardization, and calculation of methylation sites and regions (Tian *et al*., 2017). By using the ChAMP package (parameters: adjusted *P*‐value < 0.05, |deltabeta| > 0.2), differentially methylated probes (DMPs) between CC and adjacent tissue were identified. DMPs between HPV‐positive and HPV‐negative CC tissue were also identified based on the same criterion. The intersection between these two groups of DMPs was identified as HPV‐related methylation sites in the present study. Probes exhibiting deltabeta > 0.2 and adjusted *P*‐value < 0.05 were characterized as hypermethylated, and those exhibiting deltabeta < −0.2 and adjusted *P*‐value < 0.05 were characterized as hypomethylated.

### 2.3 Unsupervised hierarchical cluster analysis

To identify subtypes of CC patients, we performed unsupervised hierarchical clustering based on DNA methylation data. Clustering was performed using the beta‐values of the HPV‐related methylation sites with prognostic value.

### 2.4 Development and validation of the HPV‐related methylation signature

For further analyses, 294 patients possessing survival data were screened to investigate the relationship between DNA methylation levels and OS in CC. These 294 patients with CC were divided randomly and equally into two datasets (a training dataset and a testing dataset). The training dataset was used for identifying and establishing a prognostic signature, and the testing dataset was used for validating its predictive effectiveness (Yang *et al*., 2019). First, a univariate Cox regression analysis was performed to determine HPV‐related methylation sites with prognostic value in the training dataset. If the *P*‐value was < 0.01, the corresponding methylated sites were considered as candidate methylated sites. Through preliminary screening, there may be excess candidate methylated sites. Therefore, least absolute shrinkage and selection operator (LASSO)‐penalized Cox proportional hazards regression analysis was conducted to further reduce candidate methylated sites by using the R package ‘glmnet’ (Friedman *et al*., 2010; Wang *et al*., 2019b); LASSO is a popular algorithm that adopts explicable prediction rules and can solve the collinearity problem by dimension reduction (Gui and Li, 2005). Third, a stepwise multivariate Cox regression analysis was performed to further screen the methylated sites. An optimal predictive model was selected, similar to that used for the lowest Akaike information criterions value. The HPV‐related methylation sites in the model were utilized to establish the risk signature. The risk score was calculated using the following formula: Risk score = beta‐value of methylated site 1 × coefficient + beta‐value of methylated site 2 × coefficient + … beta‐value of methylated site *n* × coefficient. The risk score for each patient in the training dataset, testing dataset, and entire dataset was calculated based on this formula. Based on the median cutoff of the risk score, patients with CC were grouped into high‐ and low‐risk groups. Survival analysis was performed to evaluate the OS difference between high‐ and low‐risk groups that were stratified according to the signature using the ‘survival’ R package (Holleczek and Brenner, 2013). To validate the prognostic capability of this signature, we calculated area under the curve (AUC) using the ‘timeROC’ R package (Lorent *et al*., 2014). The high AUC suggested accurate predictive capability of the signature (Lorent *et al*., 2014).

### 2.5 Gene set enrichment analysis (GSEA)

To explore differences in potential biological processes in CC patients from different clusters, GSEA was performed using the hallmark gene sets (h.all.v7.0.symbols), which were obtained from the Molecular Signatures Database (https://www.gsea‐msigdb.org/gsea/msigdb/index.jsp). The hallmark gene sets display coordinate expression and represent well‐defined biological processes, providing a clearer biological space for GSEA (Halvorsen *et al*., 2019; Liberzon *et al*., 2015). The ‘fgsea’ R package was used, and 10 000 permutations were performed for each parameter analyzed to calculate the enrichment scores based on the threshold of adjusted *P*‐value < 0.05 (Sergushichev, 2016).

### 2.6 Single sample gene set enrichment analysis (ssGSEA)

The immune infiltration levels of 24 different immune cell types were estimated by performing ssGSEA in the R package ‘gsva’ (http://www.bioconductor.org/packages/release/bioc/html/GSVA.html). The marker gene set for 24 types of immune cells was obtained from a previous study (Bindea *et al*., 2013). The ssGSEA algorithm transforms marker gene expression patterns into quantities of immune cell populations in individual tumor samples (Rooney *et al*., 2015). This algorithm could identify 24 types of immune cells, including innate immune cells [natural killer (NK) cells, NK CD56dim cells, NK CD56bright cells, dendritic cells (DCs), activated DCs (aDCs), plasmacytoid DCs (pDCs), immature DCs (iDCs), neutrophils, mast cells, eosinophils, and macrophages] and adaptive immune cells [B cells, CD8+ T cells, cytotoxic cells, T cells, T helper cells, central memory T cells (Tcm cells), effector memory T cells (Tem cells), follicular helper T cells (Tfh cells), gamma delta T cells (Tgd cells), type 1T helper cells (Th1 cells), type 2T helper cells, type 17T helper cells, and regulatory T cells (Treg cells)] (Zhang *et al*., 2018).

### 2.7 Tumor microenvironments analysis

Estimation of STromal and Immune cells in MAlignant Tumor tissues using Expression data (ESTIMATE) algorithm was used to calculate immune and stromal scores to predict the infiltration of tumor microenvironment cells, by analyzing specific gene expression signature of immune and stromal cells (Yoshihara *et al*., 2013).

### 2.8 Statistical analyses

All statistical analyses were conducted by using graphpad prism 7(GraphPad Software Inc., San Diego, CA, USA) and r software (version 3.5.2, R Foundation for Statistical Computing, Vienna, Austria) unless otherwise stated. Univariate and multivariate Cox regression analyses were conducted to investigate the prognostic value of HPV‐related methylation signature and some clinicopathological variables. Volcano plots were created using R package ‘ggplot2’. Heatmaps were created using R package ‘pheatmap’. Violin plots were created using R package ‘vioplot’. Forest plots were created using R package ‘forestplot’. All statistical results with a *P*‐value < 0.05 were considered significant.

## 3 RESULTS

### 3.1 Identification and screening of HPV‐related methylation sites

**Fig. 1:**
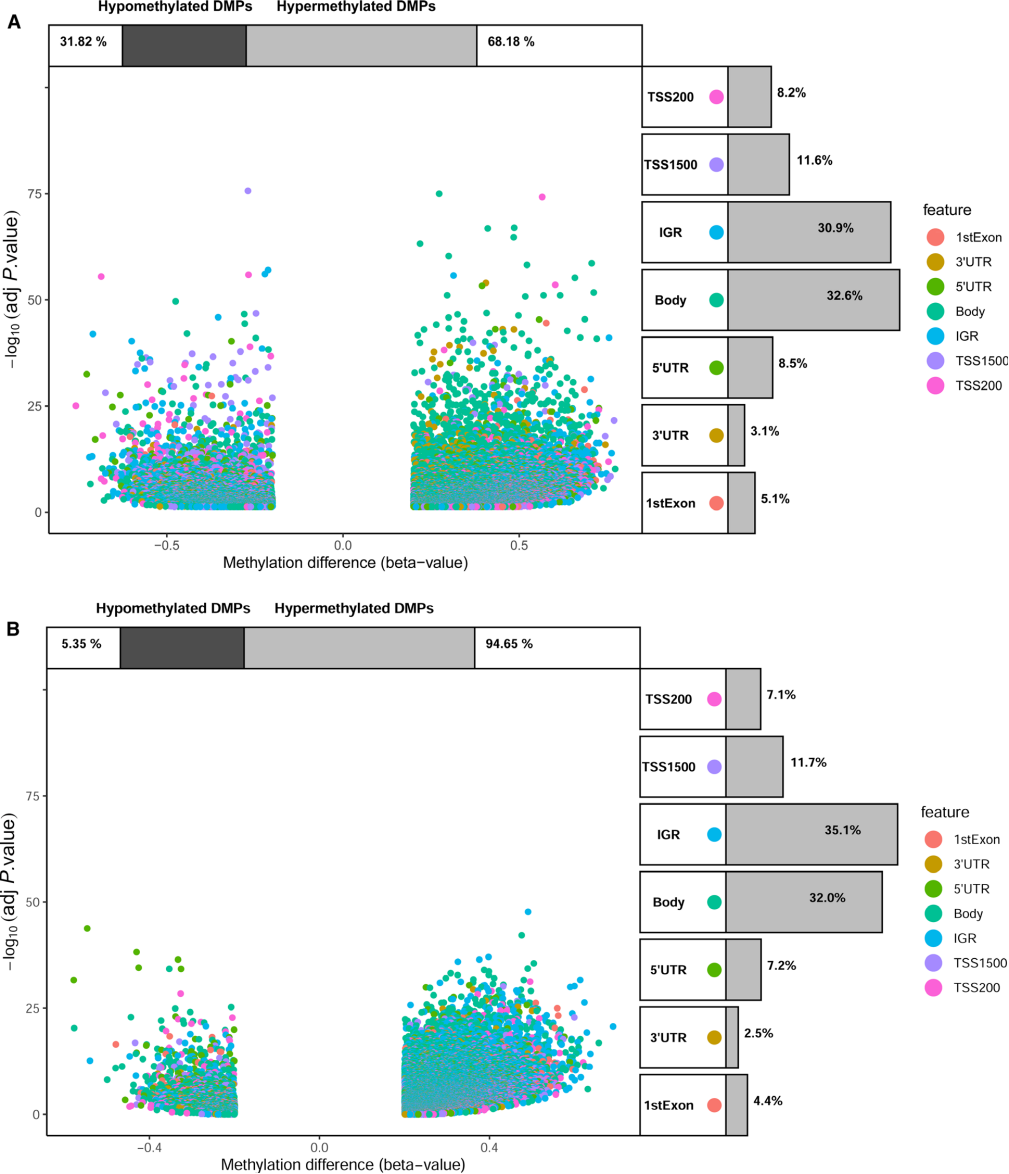
DMP analyses in cases and controls. (A) DMP analysis between CC and normal tissue. (B) DMP analysis between HPV‐positive and HPV‐negative CC tissues. Volcano plots of DMPs and position of methylation probes in relation to the gene (IGR, intergenic region; TSS, transcription start site; UTR) are displayed. The percentages of hypomethylated and hypermethylated DMPs are displayed on top. The proportions of different genomic features are shown on the right.

After removing a number of undetected methylated probes, a total of 312 samples (309 CC and three adjacent normal samples) and 372 137 DNA methylated sites were obtained from TCGA. Additionally, a total of 178 samples (169 HPV‐positive and nine HPV‐negative CC samples) and 378 494 DNA methylated sites were obtained. By performing ChAMP with the adjusted *P*‐value < 0.05 and |deltabeta| > 0.2, we identified 35 678 DMPs between tumor and normal tissue (Fig. 1A). A total of 48 190 DMPs were screened between HPV‐positive and HPV‐negative CC tissues (Fig. 1B). By intersecting these two groups of DMPs, we acquired 9249 HPV‐related methylation sites (Fig. S1). Then, 294 CC patients with survival data containing information on 9249 HPV‐related methylation sites were included in further analysis. These 294 patients were divided randomly and equally into a training dataset and a testing dataset. The clinicopathological characteristics of patients are summarized in Table S1. There were no statistically significant differences between these two groups.

### 3.2 Identification of three methylation clusters of CC exhibiting distinct survival outcomes

**Fig. 2:**
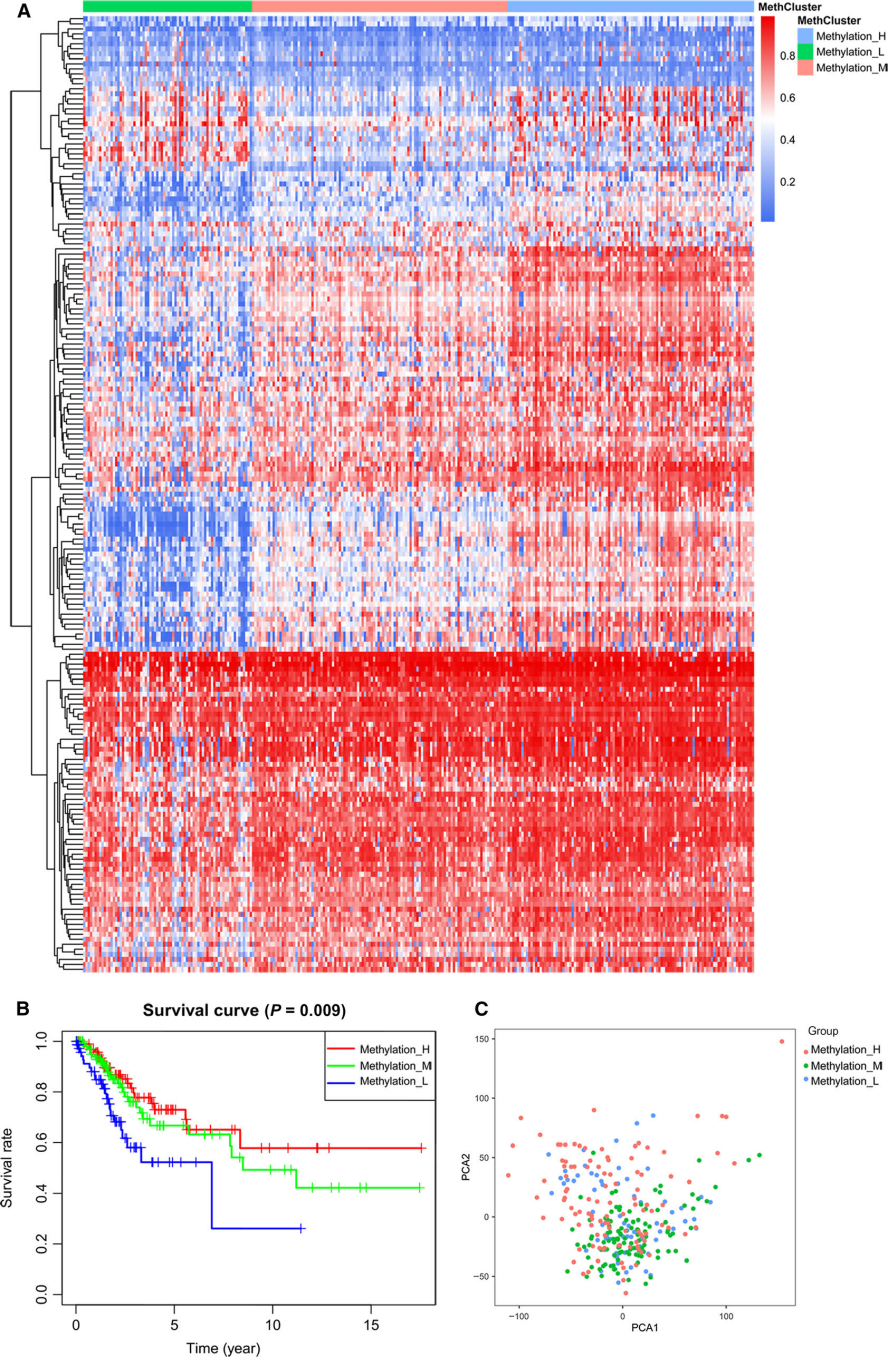
Identification of three methylation clusters of CC with distinct survival outcomes. (A) Heatmap of three methylation clusters generated by performing unsupervised hierarchical clustering. (B) Kaplan–Meier curves of OS of three methylation clusters. (C) PCA of the total RNA expression profiles in the TGGA CC dataset.

Univariate Cox regression analysis was conducted to screen DNA methylation sites that related to overall survival (OS) by using the HPV‐related methylation sites as variables in the training dataset. A total of 191 HPV‐related methylation sites that were related to OS in the training dataset (*P* < 0.05) were picked out for unsupervised clustering analysis. Consequently, 294 patients with CC were categorized into three clusters (Fig. 2A). The patients in cluster 1 (Methylation‐H) exhibited frequent hypermethylation among the 191 methylation sites. The methylation level of cluster 3 (Methylation‐L) was the lowest, and the methylation level of cluster 2 (Methylation‐M) was intermediate. Compared to Methylation‐M or Methylation‐L, Methylation‐H exhibited a significantly higher OS (*P* = 0.009, Fig. 2B). To check for cluster stability, clustering was compared for 9249 HPV‐related methylation sites (Fig. S2). The formation of clusters was robust for the 9249 HPV‐related methylation sites selected for calculation. The results demonstrated the presence of three different methylation clusters. Principal component analysis (PCA), which was further employed to compare the transcriptional profiles among the three clusters, displayed a clear distinction among these clusters. In detail, PCA showed that the samples from the three clusters were well separated from each other (Fig. 2C).

### 3.3 Biological processes and mechanisms related to different clusters of CC

**Fig. 3:**
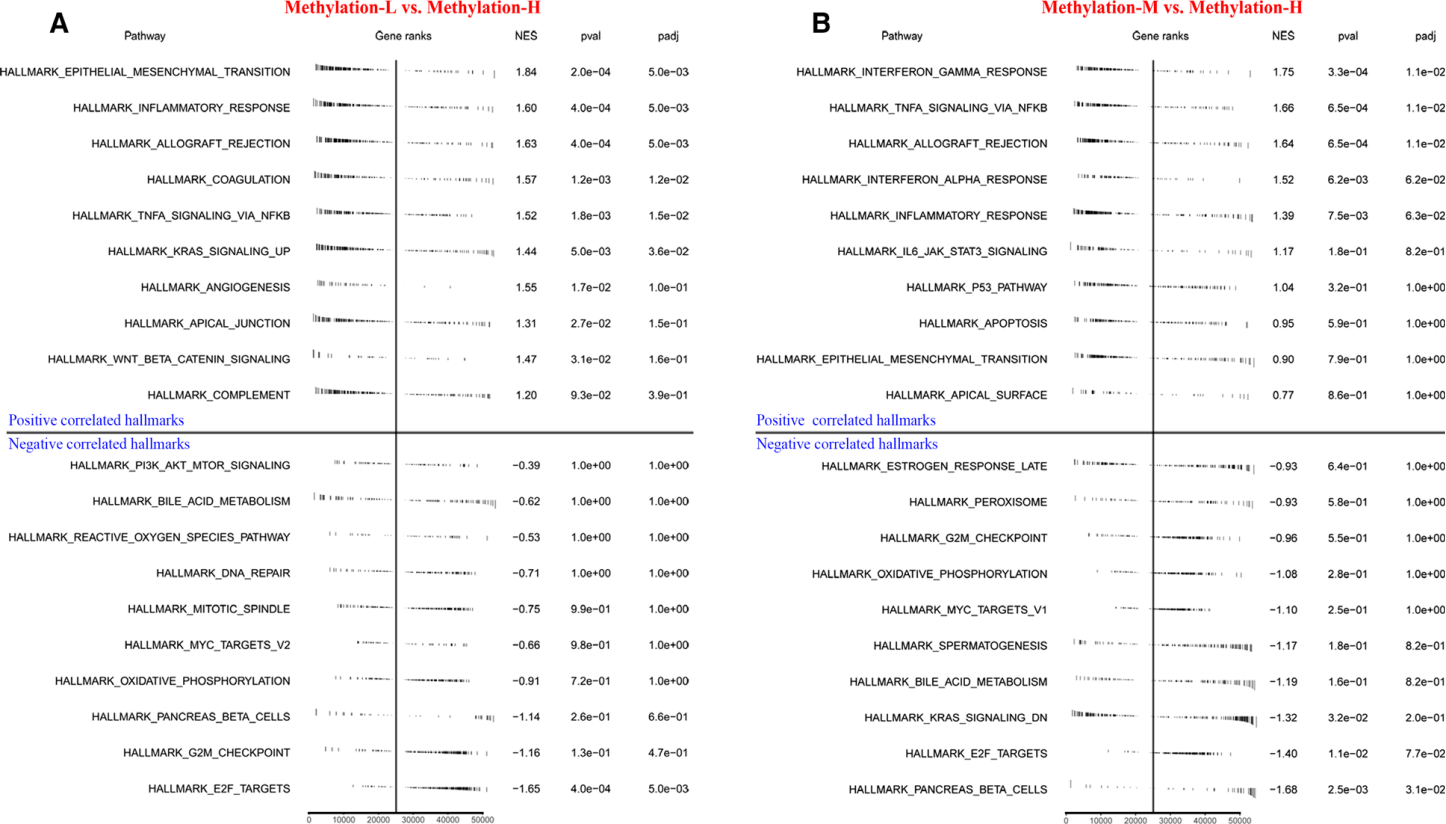
Summary of GSEA results with hallmark gene sets in Methylation‐L (A) or Methylation‐M (B), compared to Methylation‐H. Six positively enriched hallmarks and one negatively enriched hallmark with adjusted *P*‐value < 0.05 were identified in Methylation‐L. Three positively enriched hallmarks and one negatively enriched hallmark with adjusted *P*‐value < 0.05 were identified in Methylation‐M.

Gene set enrichment analysis was performed to investigate the underlying biological processes and mechanisms related to different clusters of CC. The results revealed that the hallmarks of tumors, including KRAS signaling [normalized enrichment score (NES) = 1.44, adjusted *P*‐value < 0.05], coagulation (NES = 1.57, adjusted *P*‐value < 0.05), TNFα signaling via NF‐κB (NES = 1.52, adjusted *P*‐value < 0.05), inflammatory response (NES = 1.60, adjusted *P*‐value < 0.01), and epithelial–mesenchymal transition (NES = 1.84, adjusted *P*‐value < 0.01), were significantly and positively associated with Methylation‐L (Fig. 3A). E2F targets (NES = −1.65, adjusted *P*‐value < 0.01) were significantly and negatively associated with Methylation‐L compared to Methylation‐H (Fig. 3A). Additionally, interferon‐gamma response (NES = 1.75, adjusted *P*‐value < 0.05) and TNFα signaling via NF‐κB (NES = 1.66, adjusted *P*‐value < 0.05) were significantly and positively associated with Methylation‐M compared to Methylation‐H (Fig. 3B).

### 3.4 Analysis of mutations and CNVs in CC patients within the three clusters

We further investigated the genomic alterations that could be correlated with different survival outcomes in the three clusters, with an aim to identify potential drug targets to reverse the poor prognosis of Methylation‐M and Methylation‐L.

**Fig. 4:**
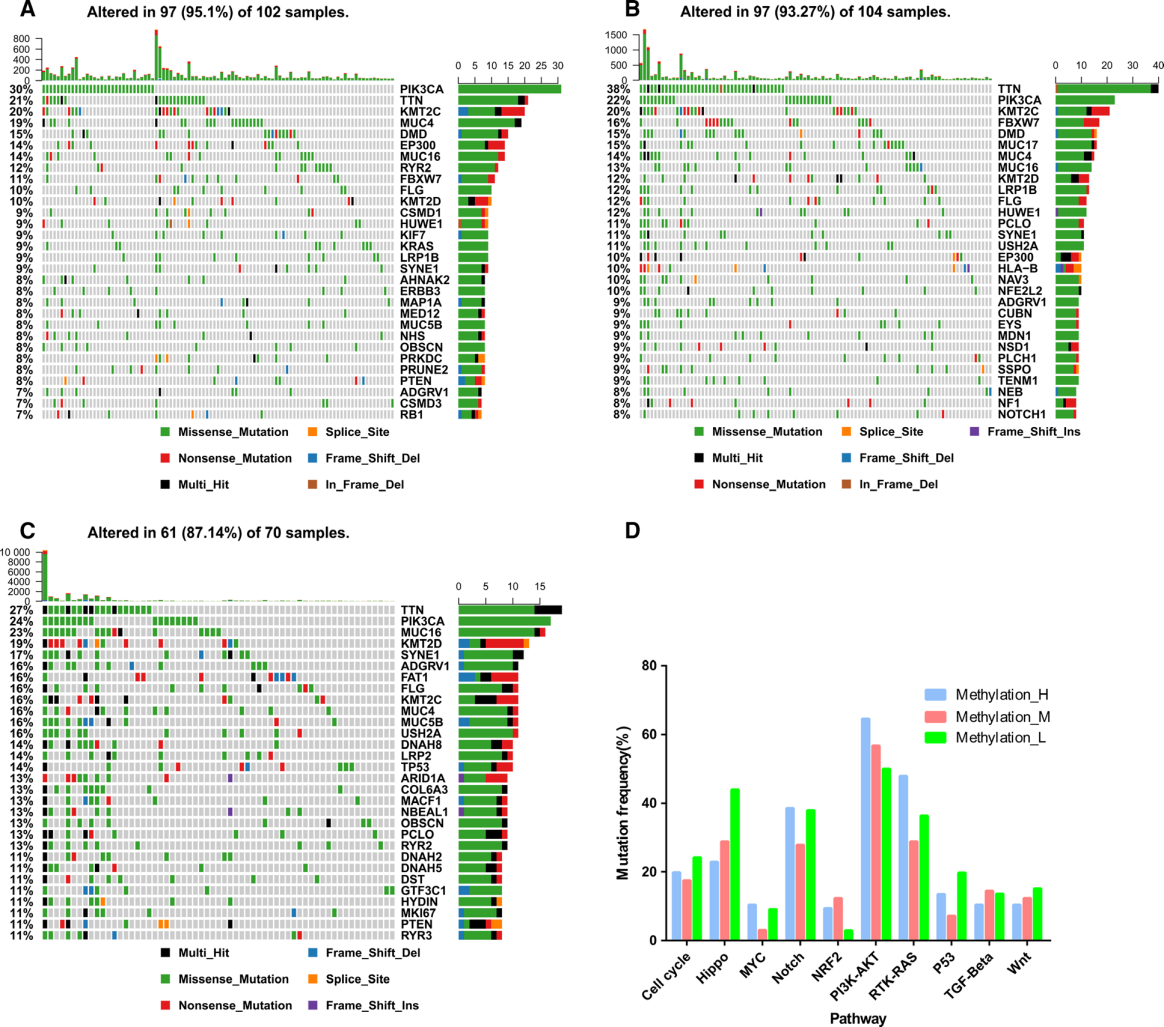
Comparison of mutations among the three methylation clusters of CC. The top 30 most frequently mutated genes in the CC patients of Methylation‐H (A), Methylation‐M (B), and Methylation‐L cluster (C). (D) The mutation frequencies of ten common oncogenic pathways among three clusters.

**Fig. 5:**
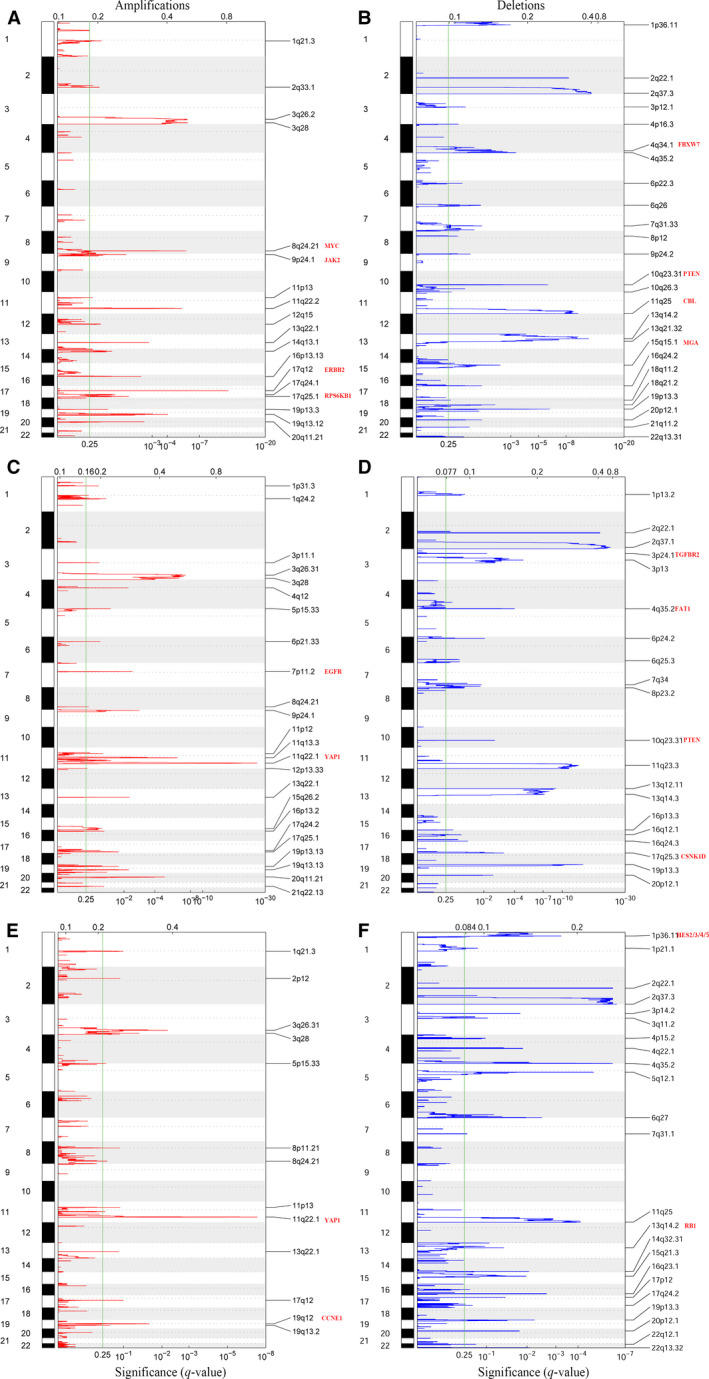
GISTIC 2.0 amplifications and deletions in Methylation‐H (A, B), Methylation‐M (C, D), and Methylation‐L (E, F) cluster. Chromosomal locations of peaks of significantly recurring focal amplifications (red) and deletions (blue) are displayed. The *q*‐values, representing the statistical significance, are displayed along the bottom. Regions with *q*‐values < 0.25 (green lines) were considered significantly altered. The locations of the peak regions of maximal copy number change and the known cancer‐related genes within those peaks are indicated to the right of each panel.

The mutation profiles in CC patients within the three methylation clusters were investigated. Among the 294 CC patients, 276 possessed available somatic mutation data. The top 30 most frequently mutated genes in the three clusters are presented in Fig. 4A–C. The mutation frequencies of 10 common oncogenic pathways among the three clusters were calculated (Figs 4D and S3–S8). Mutations among the MYC, Notch, PI3K‐AKT, and RTK‐RAS signaling pathways were most frequently detected in Methylation‐H (Figs 4D and S3–S8). The cell cycle, Hippo, P53, and Wnt signaling pathways exhibited higher mutation frequencies in Methylation‐L (Figs 4D and S3–S8). The mutation frequencies of the Hippo and TGF‐β pathways were high in Methylation‐M (Figs 4D and S3–S8). Additionally, differences in somatic CNV among the CC patients in the three clusters were evaluated using GISTIC 2.0. Among the 294 CC patients, 282 patients possessed CNV data. CNV analysis demonstrated that amplifications of 8q24.21 [*MYC* (oncogenic gene in MYC pathway)], 9p24.1 [*JAK2* (oncogenic gene in RTK‐RAS pathway)], 17q12 [*ERBB2* (oncogenic gene in RTK‐RAS pathway)], and 17q25.1 [*RPS6KB1* (oncogenic gene in PI3K‐AKT pathway)] as well as deletions of 11q25 [*CBL* (TSG in RTK‐RAS pathway)], 10q23.31 [*PTEN* (TSG in PI3K‐AKT pathway)], 4q34.1 [*FBXW7* (TSG in Notch pathway)], and 15q15.1 [*MGA* (TSG in MYC pathway)] were identified in Methylation‐H (Figs 5A,B and S9). Moreover, amplifications of 11q22.1 [*YAP1* (oncogenic gene in Hippo pathway)], 7p11.2 [*EGFR* (oncogenic gene in PI3K‐AKT pathway)], as well as deletions of 4q35.2 [*FAT1* (TSG in Hippo pathway)], 10q23.31 [*PTEN* (TSG in PI3K‐AKT pathway)], 17q25.3 [*CSNK1D* (TSG in Hippo pathway)], and 3p24.1 [*TGFBR2* (TSG in TGF‐β pathway)] were identified in Methylation‐M (Figs 5C,D and S9). Finally, amplifications of 11q22.1 [*YAP1* (oncogenic gene in Hippo pathway)] and 19q12 [*CCNE1* (oncogenic gene in cell cycle pathway)] and deletions of 13q14.2 [*RB1* (TSG in cell cycle pathway)] and 1p36.11 [*HES2/3/4/5* (TSGs in Notch pathway)] were identified in Methylation‐L (Figs 5E,F and S9).

### 3.5 Construction of the HPV‐related methylation signature

Univariate Cox regression analysis identified 20 HPV‐related methylation sites with prognostic significance in the training dataset (*P* < 0.01, Fig. S10a). After LASSO regression analysis, 11 of the 20 HPV‐related methylation sites were selected (Fig. S10b). Subsequently, a stepwise multivariate Cox regression analysis was performed for these 11 methylation sites. Finally, six methylation sites (cg23170347, cg16376000, cg13759702, cg01727408, cg05008070, and cg07227049) were identified to construct the optimal prognostic model (Fig. S10c). The risk score formula based on the DNA methylation levels and coefficients of these six HPV‐related methylation sites was calculated as follows: Risk score = 1.9939 × beta‐value of cg13759702 − 1.6941 × beta‐value of cg23170347 − 1.5290 × beta‐value of cg16376000 − 3.9910 × beta‐value of cg01727408 − 2.4146 × beta‐value of cg05008070 − 4.8805 × beta‐value of cg07227049. The DNA methylation level of cg13759702 was correlated with high risk, while the DNA methylation levels of cg23170347, cg16376000, cg01727408, cg05008070, and cg07227049 were correlated with low risk. The genes corresponding to five methylation sites (cg05008070, cg07227049, cg13759702, cg16376000, and cg23170347) were Disheveled Binding Antagonist of Beta Catenin 1 (*DACT1*), VRK Serine/Threonine Kinase 2 (*VRK2*), Melanotransferrin (*MELTF*), Fibroblast Growth Factor 12 (*FGF12*), and Prickle Planar Cell Polarity Protein 2 (*PRICKLE2*); all of these genes were protein coding genes. The list of the six HPV‐related methylation sites, their chromosomal locations, *P*‐values, and the coefficients obtained in multivariate Cox regression analysis are provided in Table 1. Pearson’s correlation test was conducted to measure the correlations between the expression of the above five protein coding genes and the methylation levels of the corresponding methylation sites (Fig. S11). Significant and negative correlations were observed between the expression of the five genes and the methylation level of corresponding methylation sites (*P* < 0.001, *R* < −0.10, Fig. S11).

**Table 1:** Six HPV‐related methylation sites in the signature. NA, not available.

Probe ID	Chromosomal location	Gene symbol	Gene type	CGI coordinate	Feature type	Coefficient^a^	*P* value^a^
cg01727408	chr16: 85575465–85575466	NA	NA	chr16:85586333‐85586656	NA	−3.9910	0.0007
cg05008070	chr14: 58639944–58639945	*DACT1*	Protein coding	chr14:58637581‐58638859	S_Shore	−2.4146	0.0599
cg07227049	chr2: 58107873–58107874	*VRK2*	Protein coding	chr2:58046507‐58047287	NA	−4.8805	< 0.0001
cg13759702	chr3: 197001599–197001600	*MELTF*	Protein coding	chr3:197002087‐197004007	N_Shore	1.9939	0.0349
cg16376000	chr3: 192409541–192409542	*FGF12*	Protein coding	chr3:192408032‐192410205	Island	−1.5290	0.0241
cg23170347	chr3: 64268119–64268120	*PRICKLE2*	Protein coding	chr3:64267857‐64268143	Island	−1.6941	0.0958

^a^ In multivariate Cox regression analysis.

### 3.6 Evaluating the predictive capability of the HPV‐related methylation signature

**Fig. 6:**
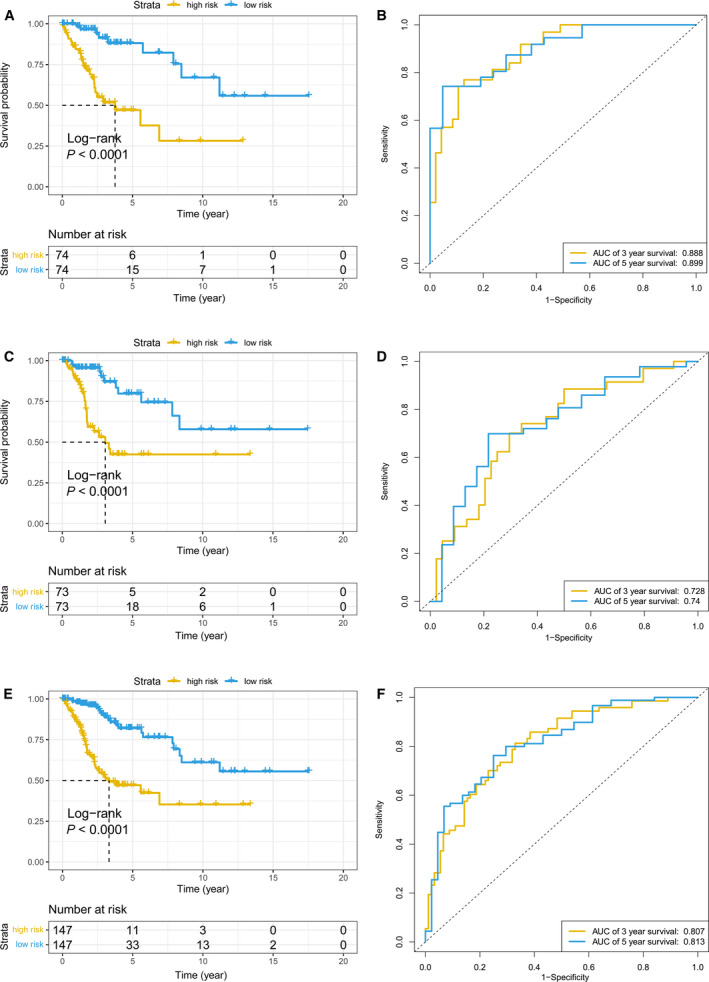
The prognostic role of the HPV‐related methylation signature in the training, testing, and entire datasets. Kaplan–Meier OS curves for patients assigned to high‐ and low‐risk groups based on the risk score in the training (A), testing (C), and entire datasets (E) are shown. Time‐dependent ROC curves in the training (B), testing (D), and entire datasets (F) are displayed.

Kaplan–Meier survival analyses were conducted in the training and testing datasets to evaluate the predictive capability of our HPV‐related methylation signature. In the training dataset, CC patients were stratified into either high‐risk (*n* = 74) or low‐risk (*n* = 74) group. These two groups possessed distinct survival outcomes (*P* < 0.0001, Fig. 6A). Low‐risk patients exhibited better OS compared to high‐risk patients. The 5‐year AUC for the six‐DNA methylation signature was 0.899; the 3‐year AUC was 0.888 (Fig. 6B). A similar result was observed in the testing dataset and the entire dataset. The risk score was calculated for patients in the testing dataset and the entire dataset, and then, each patient was marked as high risk or low risk, as previously described (Yang *et al*., 2019). There were 73 high‐risk patients and 73 low‐risk patients within the testing dataset. The survival for low‐risk patients was improved than that for the high‐risk patients (Fig. 6C, *P* < 0.0001). The AUC at 5 years was 0.74, and the 3‐year AUC was 0.728 in the testing dataset (Fig. 6D). There were 147 high‐risk patients and 147 low‐risk patients in the entire dataset. The low‐risk patients exhibited longer median survival than the high‐risk patients (2.50 vs. 1.48 years, *P* < 0.0001, Fig. 6E). In the entire dataset, the 5‐year AUC was 0.813, and the 3‐year AUC was 0.807 (Fig. 6F). The distribution of risk score, survival status, and heatmap of the six methylation sites for patients with CC in the training, testing, and entire datasets are displayed in Figs S12‐S14. Furthermore, the DNA methylation levels of the six methylation sites in the high‐ and low‐risk patients in the entire dataset were measured. We found that high‐risk patients possessed significantly higher methylation levels for cg13759702 and significantly lower methylation levels for the other four methylation sites, with the exception of cg01727408, in the entire dataset (Fig. S15, *P* < 0.001).

### 3.7 Independence of the HPV‐related methylation signature in the OS prediction from clinicopathological factors

Univariate (Fig. S16a) and multivariate Cox regression analyses (Fig. S16b) were carried out to explore if the HPV‐related methylation signature was an independent predictive indicator for the OS of CC patients. The results were adjusted for certain clinicopathological variables including age, grade, pathologic stage, clinical stage, and tumor status. The sample size was small after we excluded samples with unknown M stage (*n* = 169, >50%) and unknown HPV status (*n* = 118, 40.14%); therefore, M stage and HPV status were not included in the univariate and multivariable models. As a result, our signature could serve as an independent prognostic indicator within the entire dataset in the multivariate analysis (HR (95% CI) = 1.096(1.037–1.159), *P* = 0.001, Fig. S16b). To assess the independence of this HPV‐related methylation signature, CC patients were reclassified according to different clinicopathological characteristics (Table 2). The results revealed that the signature was independent of age, clinical stage, histologic grade, T stage, lymph node metastasis, and tumor status; the signature was effective to stratify the prognosis of patients with CC.

**Table 2:** Results of Kaplan–Meier and ROC analyses based on different subgroups. ROC, receiver operating characteristic.

Variables	Group	Sample size	Kaplan–Meier, *P* value	3‐year AUC	5‐year AUC
Age(years)	≤ 50	182	< 0.0001	0.815	0.778
	> 50	112	< 0.0001	0.806	0.859
Clinical stage	I/II	225	< 0.0001	0.790	0.814
	III/IV	63	0.0007	0.851	0.831
Histologic grade	G1/2	148	< 0.0001	0.785	0.823
	G3/4	119	0.0023	0.838	0.824
T stage	T1	169	0.0012	0.800	0.760
	T2/3/4	104	< 0.0001	0.845	0.936
Lymph node metastasis	No	164	0.0002	0.814	0.773
	Yes	64	0.0180	0.795	0.789
Tumor status	Tumor free	199	0.0340	0.838	0.789
	With tumor	80	0.0004	0.776	0.903

### 3.8 Immune landscape of cervical cancer patients within different subgroups

**Fig. 7:**
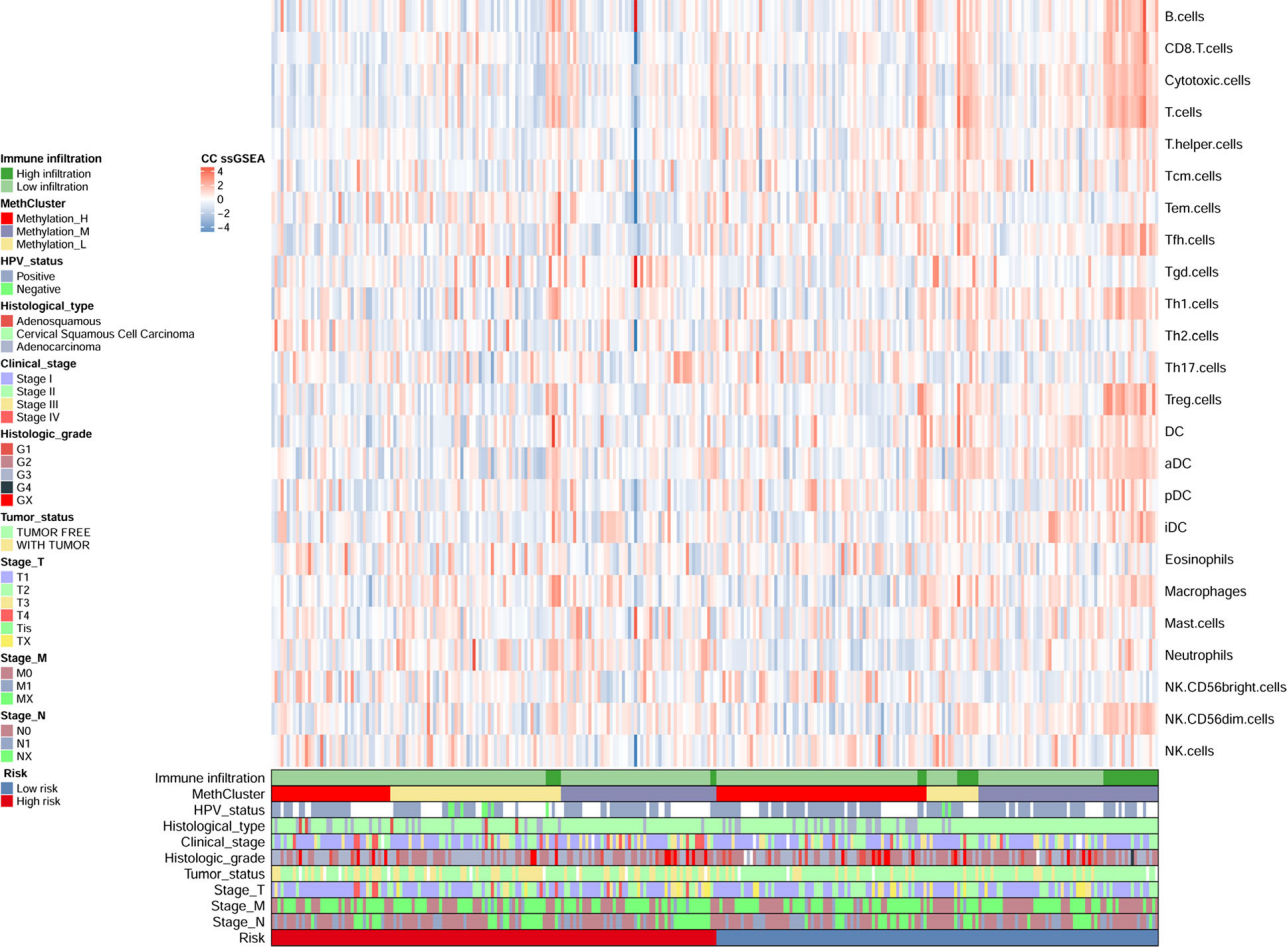
Immune landscape of CC patients within different subgroups. The heatmap showed single sample GSEA scores from 24 immune cell types of 294 patients from TCGA. HPV status, clinical stage, tumor status, histologic grade, T/N/M stage, histological type, methylation cluster, and risk were annotated in the lower panel. Hierarchical clustering was performed with Euclidean distance and Ward linkage. Two distinct immune infiltration clusters, here termed high infiltration and low infiltration, were defined.

To compare the differences in the proportions of 24 immune cells between CC low‐ and high‐risk patients and to explore the heterogeneity of immune infiltration in CC of the three methylation clusters, ssGSEA was conducted to estimate the relative proportion of the 24 immune cells in individual CC patient. The heatmap revealed the tumor‐infiltrating immune‐cell landscape of 294 CC patients from TCGA (Fig. 7).

Among the 13 adaptive immune cell types, the low‐risk group possessed significantly high proportions of B cells, CD8 T cells, cytotoxic cells, T cells, T helper cells, Tcm cells, Tfh cells, Th1 cells, and Treg cells (*P* < 0.05, Fig. S17a) compared to those of the high‐risk group. Among the 11 innate immune cell types, the low‐risk group possessed significantly higher proportions of DCs, aDCs, pDCs, iDCs, and neutrophils compared to those of the high‐risk group (*P* < 0.05, Fig. S17b). Additionally, Methylation‐H was characterized by relatively low infiltration of adaptive immune cells and innate immune cells, including CD8 T cells, cytotoxic cells, T cells, Tem cells, Tfh cells, Tgd cells, Th1 cells, Treg cells, DCs, aDCs, iDCs, neutrophils, and macrophages (*P* < 0.05, Fig. S18). Consistent with this, the analysis of tumor microenvironments demonstrated that Methylation‐H exhibited a lower ESTIMATE score, immune score, stromal score, and a higher tumor purity compared to the other two clusters (*P* < 0.05, Fig. S19). These results indicated that compared to the other two clusters, Methylation‐H possessed a different immune phenotype that was featured by less immune infiltration and lower immune activation.

## 4 Discussion

Accurate subtype identification, prognostic stratification, and characterization of the underlying mechanism are crucial for our understanding of cancers and for the guidance of treatment management and personalized therapies. DNA methylation profiling is a recent method that has been used to improve tumor classification, and this technique has already led to re‐definitions and sub‐classifications of various tumors such as glioblastoma (Noushmehr *et al*., 2010), head and neck squamous carcinoma (Brennan *et al*., 2017), adrenocortical carcinoma (Barreau *et al*., 2013), and hepatocellular carcinoma (Li *et al*., 2019). Furthermore, several prognostic signatures based on DNA methylation sites have been reported to stratify the prognosis of various cancers such as cutaneous melanoma (Guo *et al*., 2019), ovarian cancer (Guo *et al*., 2018), lung adenocarcinoma (Wang *et al*., 2019a), and breast cancer (Tao *et al*., 2019). These studies support that DNA methylation sites can serve as promising biomarkers for subtype identification and prognostic stratification in cancer. HPV infection is a key oncogenic driver in CC. HPV infection causes epigenetic reprogramming of the host cell during malignant transformation, subsequently resulting in distinct HPV‐related epigenetic phenotypes. Therefore, this study was intended to determine the epigenetic alterations involved in CC progression and HPV infection. Additionally, HPV‐related DNA methylation signatures were explored to identify the different CC subtypes in patients and to stratify the prognosis of CC.

### 4.1 Subtype identification

Based on the HPV‐related methylation sites, CC patients were classified into three heterogeneous clusters. Compared to Methylation‐M and Methylation‐L, Methylation‐H exhibited a significantly improved OS. The hallmarks of tumors, including KRAS signaling, TNFα signaling via NF‐κB, inflammatory response, epithelial–mesenchymal transition, and interferon‐gamma response, were enriched in Methylation‐M and Methylation‐L. A great deal of research work has suggested that these biological processes or pathways play a significant role in tumorigenesis and the progression of CC (Kang *et al*., 2007; Kloth *et al*., 2005; Lages *et al*., 2011; Lee *et al*., 2008). We reasoned that the HPV‐related methylation signature might take an important part in CC via the above biological processes or pathways. Based on mutation and CNV analyses, we found that mutations among the MYC, Notch, PI3K‐AKT, and RTK‐RAS pathways were most frequently detected in Methylation‐H. Concurrently, the amplifications of oncogenes, such as *JAK2* and *ERBB2* in the RTK‐RAS pathway and *RPS6KB1* in the PI3K‐AKT pathway, and the deletions of TSGs, such as *CBL* in the RTK‐RAS pathway and *PTEN* in the PI3K‐AKT pathway, were identified in Methylation‐H. Therefore, we speculated that hyper‐activated RTK‐RAS or PI3K‐AKT pathways in tumor may take an important part in Methylation‐H. The mutation frequencies of the Hippo and TGF‐β pathways were high in Methylation‐M. The amplifications of oncogenic genes, such as *YAP1* within the Hippo pathway and *EGFR* within the PI3K‐AKT pathway, and the deletions of TSGs, such as *FAT1* and *CSNK1D* within the Hippo pathway, *TGFBR2* within the TGF‐β pathway, and *PTEN* within the PI3K‐AKT pathway, were identified in Methylation‐M. Moreover, the cell cycle and Hippo signaling pathways exhibited higher mutation frequencies in Methylation‐L. The amplifications of oncogenic genes, such as *YAP1* within the Hippo pathway and *CCNE1* within the cell cycle pathway, and the deletions of TSGs, such as *RB1* within the cell cycle pathway, were identified in Methylation‐L. Therefore, we speculated that the Hippo, TGF‐β, and cell cycle pathways might be responsible for the poor outcome observed in Methylation‐M and Methylation‐L. Overall, our analyses revealed that certain biological processes, pathways, and genomic alterations may result in a worse OS in Methylation‐L and Methylation‐M. Future studies are required to elucidate the role of these biological processes, pathways, and genomic alterations in HPV‐related epigenetic phenotypes that specifically drive cancer development.

### 4.2 Risk stratification

We constructed and verified a prognostic risk signature using six HPV‐related methylation sites (cg01727408, cg05008070, cg07227049, cg13759702, cg16376000, and cg23170347) that stratified CC patients into high‐ and low‐risk groups. The genes corresponding to the five methylation sites (cg05008070, cg07227049, cg13759702, cg16376000, and cg23170347) were *DACT1*, *VRK2*, *MELTF*, *FGF12*, and *PRICKLE2*, which were all protein coding genes. *DACT1*, *VRK2*, *MELTF*, and *FGF12* have been implicated in cancers other than CC (Dmitriev *et al*., 2015; Fernandez *et al*., 2010; Guo *et al*., 2017), and *PRICKLE2* has been reported to correlate with CC (Senchenko *et al*., 2013). Future studies will be required to elucidate the functional impact of aberrant methylation of these five genes in CC development. The corresponding encoded proteins affected by aberrant methylation may also represent promising drug targets for cancer therapy.

Our HPV‐related methylation signature could still act as an independent prognostic predictor, after adjusting for certain clinicopathological variables. Subgroup analyses further highlighted that the signature possessed strong and independent predictive power when CC patients were regrouped according to different clinicopathological characteristics. Additionally, this signature possessed higher predictive performance for patients in advanced T and clinical stages (3‐year AUCs were 0.845 and 0.851, respectively). Based on this, combining this signature with other clinical factors could serve as a promising tool for the prognosis of CC patients.

Finally, GSEA further revealed the connection between the signature and immune systems. Therefore, ssGSEA was conducted to estimate the relative proportion of the 24 immune cells in individual patient with CC. We aimed to compare the differences in the proportions of 24 immune cells between low‐risk and high‐risk patients with CC and to explore the heterogeneity of immune infiltration in CC within the three methylation clusters. Consequently, the low‐risk group possessed a significantly higher proportion of immune cells that were involved in adaptive and innate immune responses compared to that of the high‐risk group. Contrary to expectations, Methylation‐H was characterized by relatively low infiltration of adaptive immune cells and innate immune cells.

### 4.3 Strength and limitations

To our knowledge, this is the first study to explore HPV‐related DNA methylation signatures to identify the different subtypes of CC and to stratify the prognosis of CC. The molecular differences between the identified subtypes may allow these subtypes to be targeted separately under specific therapeutic approaches. In terms of clinical utility, a novel risk signature based on six HPV‐related methylation sites was identified and verified. This signature can be tested as a prognostic tool to determine patients at high risk with the potential for multimodal therapy.

This study has a few limitations. Firstly, the sample size containing information with HPV status and HPV subtypes was relatively small, and we were unable to explore the association between the HPV‐related methylation signature and HPV subtypes. Secondly, an ideal prognostic signature is one that can also efficiently risk‐stratify in other independent datasets, and we could not yet locate another dataset to further validate the performance of our six‐DNA methylation signature. Lastly, the TCGA dataset enrolled for analysis was primarily collected from patients with CC in Western countries and lacked data from Asian countries.

## 5 Conclusions

In conclusion, our study revealed that CC patients could be classified into three heterogeneous clusters based on the HPV‐related methylation sites. Specific biological processes, pathways, and genomic alterations could be correlated with the different outcomes in the three clusters. Additionally, we derived a prognostic risk signature using six HPV‐related methylation sites that stratified the patients with CC into high‐ and low‐risk groups. This study provides new insight into epigenetic biomarkers that could help to improve subtype identification, risk stratification, and treatment management.

## Conflict of interest

The authors declare no conflict of interest.

## Author contributions

SY and YW collected and analyzed the data, and wrote the manuscript. SQW, PX, and YJD analyzed the data and reviewed the manuscript. MW, KL, and TT participated in analyzing the data. YYZ, NL, and LHZ participated in preparation of the figures and tables. ZJD and HFK designed the study and revised the manuscript. All the authors read and approved the final manuscript.

## Ethics approval

Not applicable.

## Consent for publication

Not applicable.

## Supporting information


**Fig. S1.** Venn diagram for the intersections between 48 190 DMPs (HPV‐positive vs. HPV‐negative) and 35 678 DMPs (tumor vs. normal). vs.: versus; DMPs: differentially methylated probes.
**Fig. S2.** Unsupervised clustering analysis of 9249 HPV‐related methylation sites.
**Fig. S3.** Comparison of mutations among the three methylation clusters of cervical cancer. The mutation frequencies of the cell cycle (a), MYC (b), NRF2 (c), TGF‐β (d), and P53 (e) signaling pathways among the three clusters are shown.
**Fig. S4.** The mutation frequencies of the Hippo signaling pathway among the three clusters are shown.
**Fig. S5.** The mutation frequencies of the Notch signaling pathway among the three clusters are shown.
**Fig. S6.** The mutation frequencies of the RTK‐RAS signaling pathway among the three clusters are shown.
**Fig. S7.** The mutation frequencies of the PI3K‐AKT signaling pathway among the three clusters are shown.
**Fig. S8.** The mutation frequencies of the Wnt signaling pathway among the three clusters are shown.
**Fig. S9.** Comparison of copy number variations among three methylation clusters of cervical cancer. (a) Copy number gistic score for Methylation‐H, Methylation‐M, and Methylation‐L cluster. (b) Copy number frequency for Methylation‐H, Methylation‐M, and Methylation‐L cluster. Copy number gistic score/copy number frequency is indicated on the y‐axis and chromosome on the x‐axis. Individual chromosomes are separated by dotted lines with ‘red’ indicating copy number gain and ‘blue’ indicating copy number loss.
**Fig. S10.** The process of developing a prognostic signature containing six HPV‐related methylation sites. The hazard ratios (HR), 95% confidence intervals (CI) calculated by univariate Cox regression (a), the results of LASSO regression (b), and the coefficients calculated by multivariate Cox regression analysis (c) are shown.
**Fig. S11.** Association between the expression of five genes and the methylation levels of the corresponding methylation sites. Level of gene expression is reported as log_2_‐transformed FPKM, and the methylation levels of methylation sites were beta‐values.
**Fig. S12.** The distribution of risk score, survival status, and the heatmap of six methylation sites for cervical cancer patients in the training dataset.
**Fig. S13.** The distribution of risk score, survival status, and the heatmap of six methylation sites for cervical cancer patients in the testing dataset.
**Fig. S14.** The distribution of risk score, survival status, and the heatmap of six methylation sites for cervical cancer patients in the entire dataset.
**Fig. S15.** Boxplots of beta‐value in samples of patients in high‐ and low‐risk groups in the entire dataset. Mann–Whitney U test was used to determine the differences between the two groups.
**Fig. S16.** Univariate and multivariate Cox regression analyses of the association between clinicopathological factors, risk score and overall survival of patients in the TCGA cervical cancer dataset.
**Fig. S17**. The relative abundance of the 24 immune cells types in cervical cancer high‐risk and low‐risk groups. A green violin represents the low‐risk group. A red violin represents the high‐risk group. The white points inside the violin represent median values. Wilcoxon test was implemented to evaluate the differences in infiltration levels of the 24 immune cell types between the two groups.
**Fig. S18**. The relative abundance of the 24 immune cells among the three clusters. Kruskal–Wallis test was used to determine the differences between the three clusters. ns no significance, *P < 0.05, **P < 0.01, and *** *P* < 0.001.
**Fig. S19**. The analysis of tumor microenvironments among the three clusters. (a) Distribution of ESTIMATE scores of three clusters. (b) Distribution of immune scores of three clusters. (c) Distribution of stromal scores of three clusters. (d) Distribution of tumor purity of three clusters. Kruskal–Wallis test was used to determine the differences between the three clusters. ns no significance, **P < 0.01, and *** *P* < 0.001.

**Table S1.** Clinical variables in the training and testing datasets.

**Table S2.** HPV infection status of 178 cervical cancer patients from TCGA.

## Data Availability

TCGA DNA methylation data from 309 CC samples and 3 adjacent normal samples based on the Illumina HumanMethylation 450 (450K) platform (Illumina Inc.) were downloaded from UCSC xena (https://xena.ucsc.edu/). Clinical information for 307 CC patients was obtained from UCSC xena. The RNA‐sequencing gene expression profiles for 306 CC patients, somatic mutation profiles for 289 CC patients, and CNV profiles for 297 CC patients were downloaded from TCGA data portal (https://portal.gdc.cancer.gov/). The hallmark gene sets (h.all.v7.0.symbols) were obtained from the Molecular Signatures Database (https://www.gsea‐msigdb.org/gsea/msigdb/index.jsp).
