# Supplementary material for: HPV‐related methylation‐based reclassification and risk stratification of cervical cancer
Source: Mol Oncol. 2020 Jun 2;14(9):2124–41. doi: 10.1002/1878-0261.12709 (PMC7463306; doi:10.1002/1878-0261.12709)
Supplement: Supplementary file 1 — Fig. S1. Venn diagram for the intersections between 48 190 DMPs (HPV‐positive vs. HPV‐negative) and 35 678 DMPs (tumor vs. normal). vs.: versus; DMPs: differentially methylated probes. Fig. S2. Unsupervised clustering analysis of 9249 HPV‐related methylation sites. Fig. S3. Comparison of mutations among the three methylation clusters of cervical cancer. The mutation frequencies of the cell cycle (a), MYC (b), NRF2 (c), TGF‐β (d), and P53 (e) signaling pathways among the three clusters are shown. Fig. S4. The mutation frequencies of the Hippo signaling pathway among the three clusters are shown. Fig. S5. The mutation frequencies of the Notch signaling pathway among the three clusters are shown. Fig. S6. The mutation frequencies of the RTK‐RAS signaling pathway among the three clusters are shown. Fig. S7. The mutation frequencies of the PI3K‐AKT signaling pathway among the three clusters are shown. Fig. S8. The mutation frequencies of the Wnt signaling pathway among the three clusters are shown. Fig. S9. Comparison of copy number variations among three methylation clusters of cervical cancer. (a) Copy number gistic score for Methylation‐H, Methylation‐M, and Methylation‐L cluster. (b) Copy number frequency for Methylation‐H, Methylation‐M, and Methylation‐L cluster. Copy number gistic score/copy number frequency is indicated on the y‐axis and chromosome on the x‐axis. Individual chromosomes are separated by dotted lines with ‘red’ indicating copy number gain and ‘blue’ indicating copy number loss. Fig. S10. The process of developing a prognostic signature containing six HPV‐related methylation sites. The hazard ratios (HR), 95% confidence intervals (CI) calculated by univariate Cox regression (a), the results of LASSO regression (b), and the coefficients calculated by multivariate Cox regression analysis (c) are shown. Fig. S11. Association between the expression of five genes and the methylation levels of the corresponding methylation sites. Level of gene expression [file MOL2-14-2124-s001.pdf]

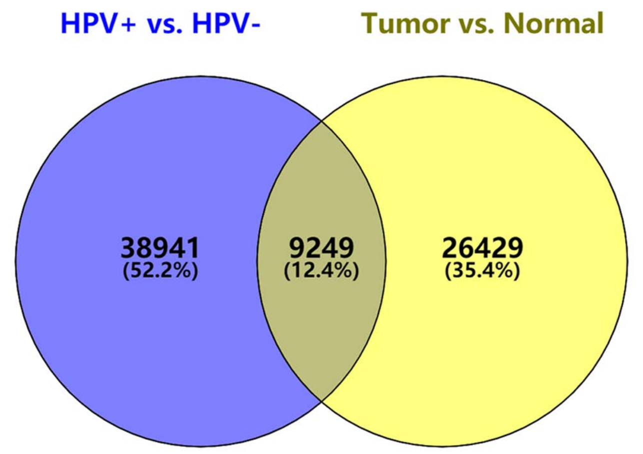
HPV+ vs. HPV-
Tumor vs. Normal
38941
(52.2%)
9249
(12.4%)
26429
(35.4%)

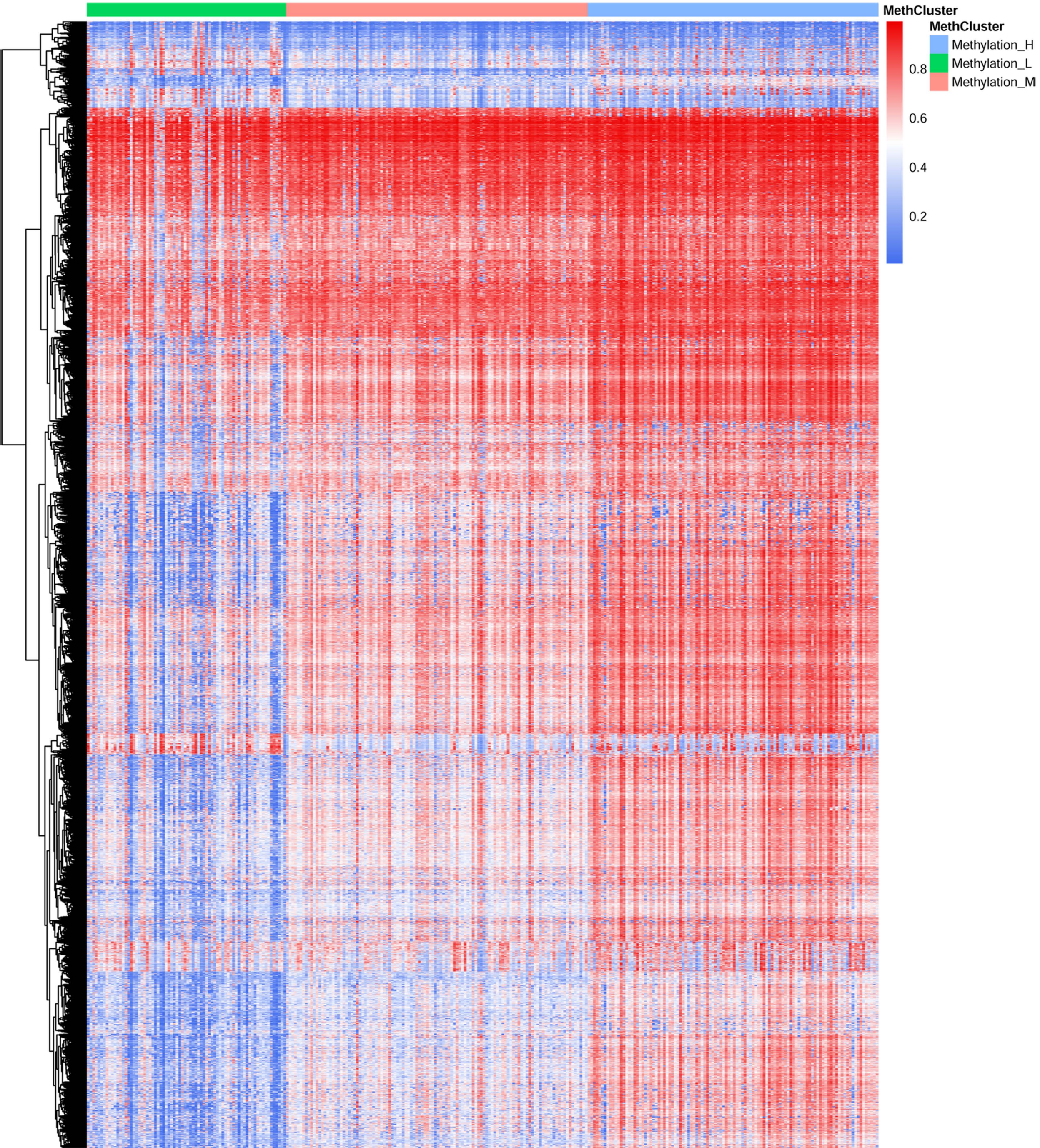
MethCluster
MethCluster
Methylation_H
Methylation_L
Methylation_M
0.8
0.6
0.4
0.2

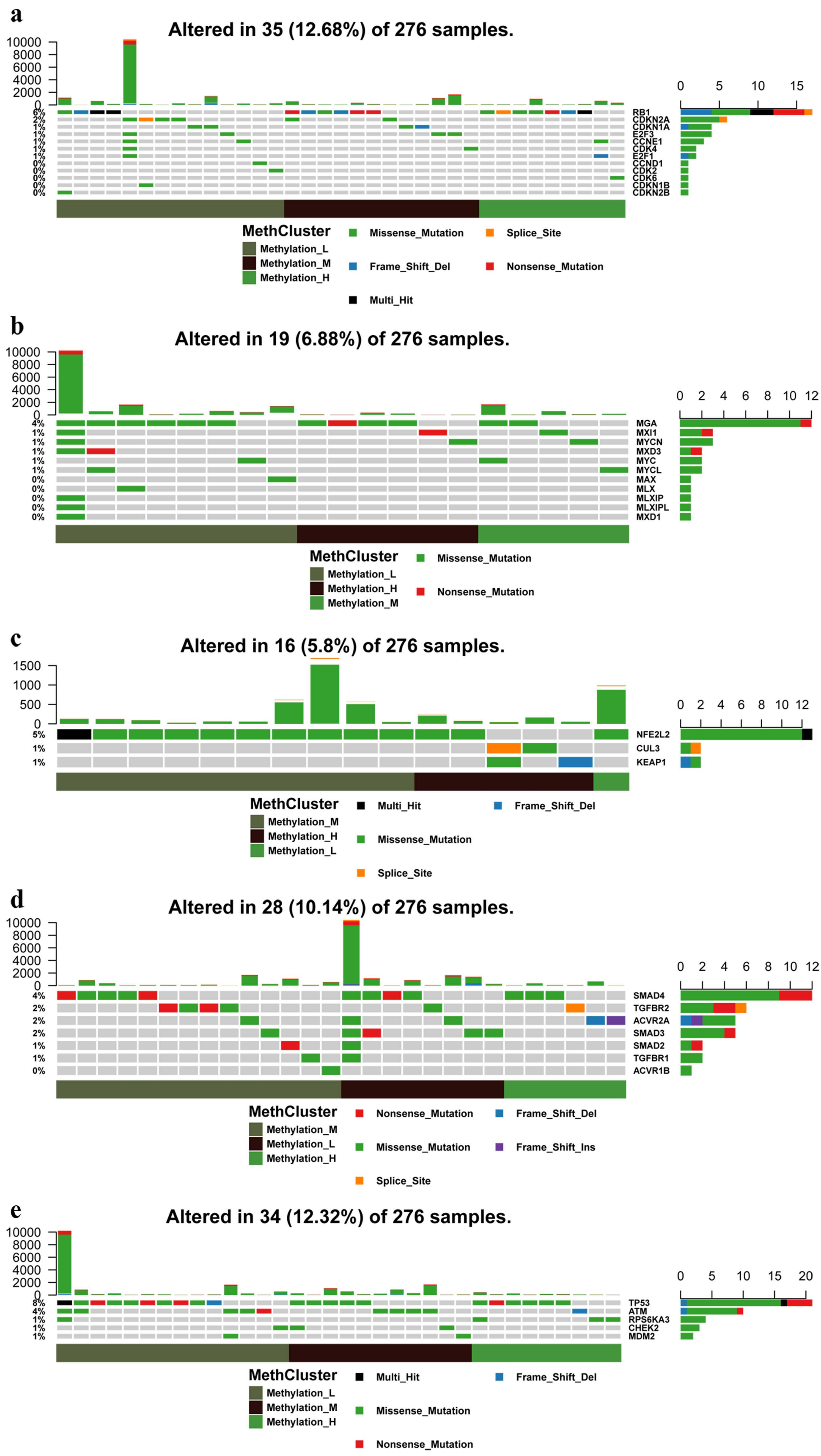
a
Altered in 35 (12.68%) of 276 samples.
RB1
CDKN2A
CDKN1A
E2F3
CCNE1
CDK4
E2F1
CCND1
CDK2
CDK6
CDKN1B
CDKN2B
MethCluster
Methylation_L
Methylation_M
Methylation_H
Missense_Mutation
Splice_Site
Frame_Shift_Del
Nonsense_Mutation
Multi_Hit
b
Altered in 19 (6.88%) of 276 samples.
MGA
MXI1
MYCN
MXD3
MYC
MYCL
MAX
MLX
MLXIP
MLXIPL
MXD1
MethCluster
Methylation_L
Methylation_H
Methylation_M
Missense_Mutation
Nonsense_Mutation
c
Altered in 16 (5.8%) of 276 samples.
NFE2L2
CUL3
KEAP1
MethCluster
Methylation_M
Methylation_H
Methylation_L
Multi_Hit
Frame_Shift_Del
Missense_Mutation
Splice_Site
d
Altered in 28 (10.14%) of 276 samples.
SMAD4
TGFBR2
ACVR2A
SMAD3
SMAD2
TGFBR1
ACVR1B
MethCluster
Methylation_M
Methylation_L
Methylation_H
Nonsense_Mutation
Frame_Shift_Del
Missense_Mutation
Frame_Shift_Ins
Splice_Site
e
Altered in 34 (12.32%) of 276 samples.
TP53
ATM
RPS6KA3
CHEK2
MDM2
MethCluster
Methylation_L
Methylation_M
Methylation_H
Multi_Hit
Frame_Shift_Del
Missense_Mutation
Nonsense_Mutation

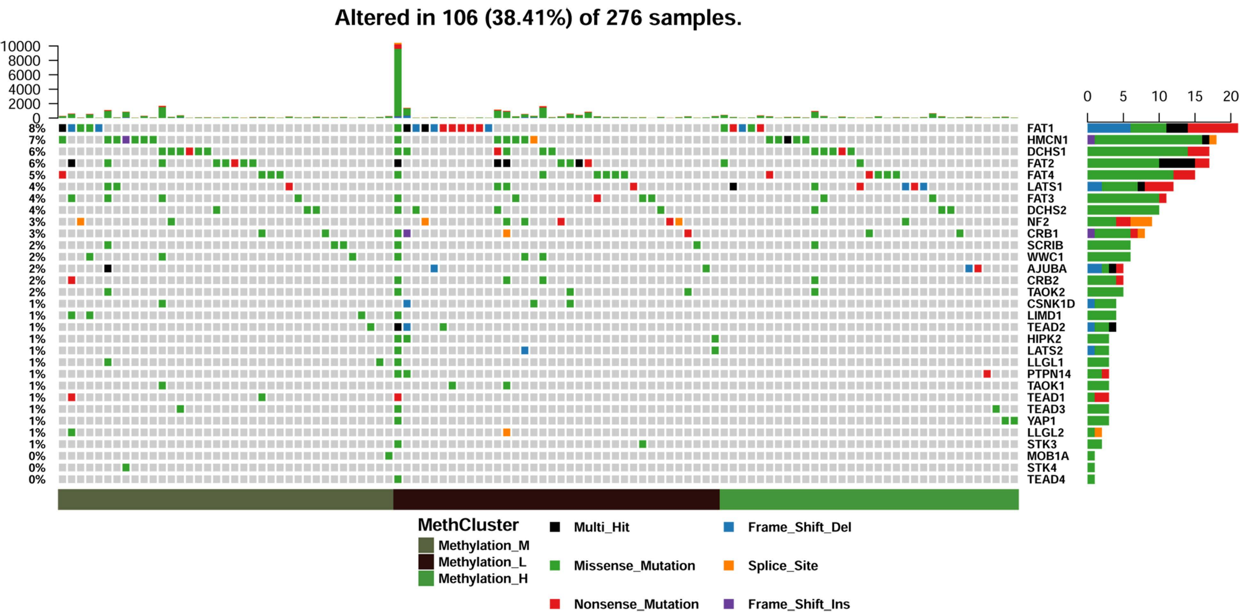
Altered in 106 (38.41%) of 276 samples.
10000
8000
6000
4000
2000
0
0
5
10
15
20
8% FAT1
7% HMCN1
6% DCHS1
6% FAT2
5% FAT4
4% LATS1
4% FAT3
4% DCHS2
3% NF2
3% CRB1
2% SCRIB
2% WWC1
2% AJUBA
2% CRB2
2% TAOK2
1% CSNK1D
1% LIMD1
1% TEAD2
1% HIPK2
1% LATS2
1% LLGL1
1% PTPN14
1% TAOK1
1% TEAD1
1% TEAD3
1% YAP1
1% LLGL2
1% STK3
0% MOB1A
0% STK4
0% TEAD4
MethCluster
Methylation_M
Methylation_L
Methylation_H
Multi_Hit
Missense_Mutation
Nonsense_Mutation
Frame_Shift_Del
Splice_Site
Frame_Shift_Ins

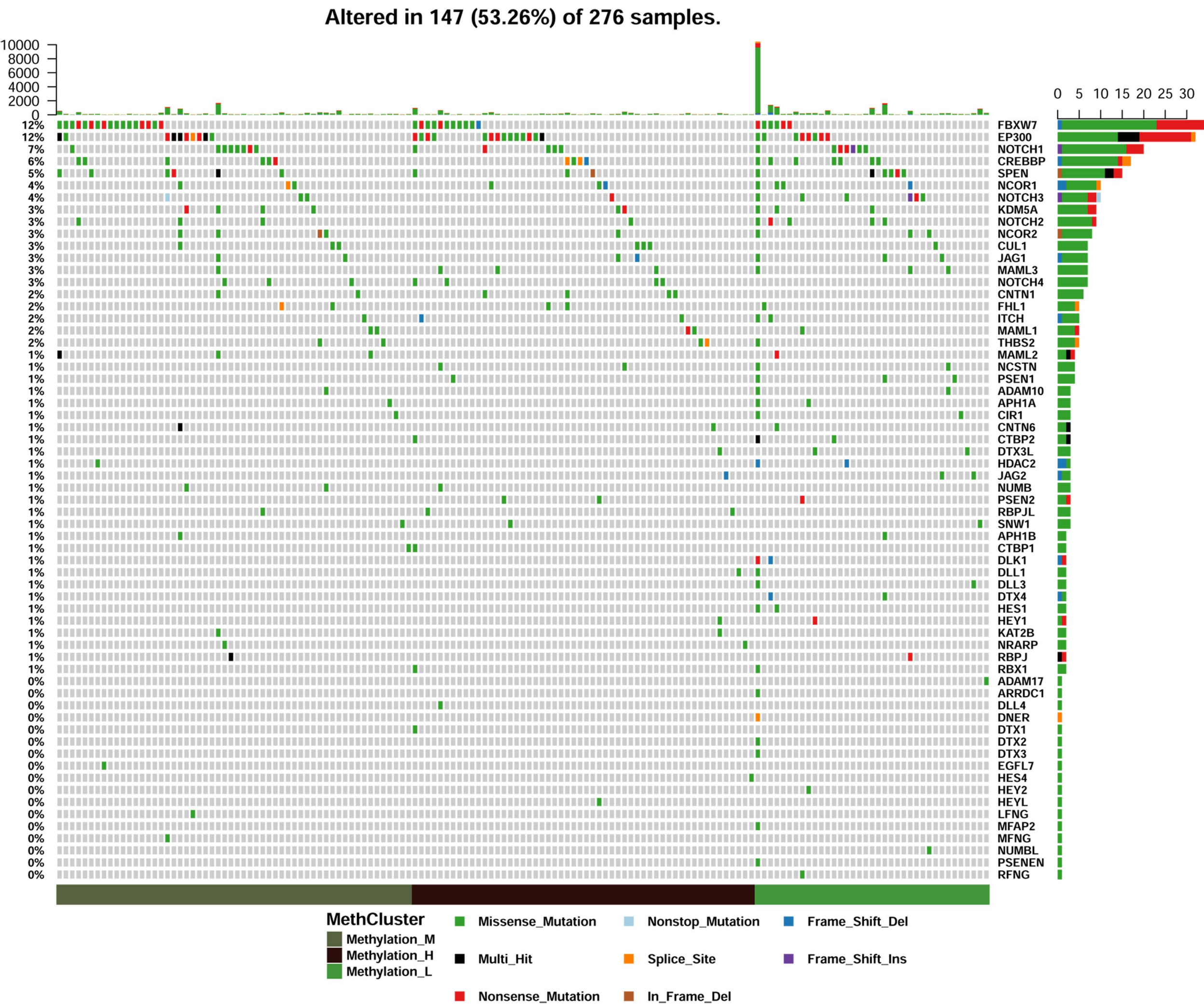

Altered in 147 (53.26%) of 276 samples.
10000
8000
6000
4000
2000
0
0 5 10 15 20 25 30
12% FBXW7
12% EP300
7% NOTCH1
6% CREBBP
5% SPEN
4% NCOR1
4% NOTCH3
3% KDM5A
3% NOTCH2
3% NCOR2
3% CUL1
3% JAG1
3% MAML3
3% NOTCH4
2% CNTN1
2% FHL1
2% ITCH
2% MAML1
2% THBS2
1% MAML2
1% NCSTN
1% PSEN1
1% ADAM10
1% APH1A
1% CIR1
1% CNTN6
1% CTBP2
1% DTX3L
1% HDAC2
1% JAG2
1% NUMB
1% PSEN2
1% RBPJL
1% SNW1
1% APH1B
1% CTBP1
1% DLK1
1% DLL1
1% DLL3
1% DTX4
1% HES1
1% HEY1
1% KAT2B
1% NRARP
1% RBPJ
1% RBX1
0% ADAM17
0% ARRDC1
0% DLL4
0% DNER
0% DTX1
0% DTX2
0% DTX3
0% EGFL7
0% HES4
0% HEY2
0% HEYL
0% LFNG
0% MFAP2
0% MFNG
0% NUMBL
0% PSENEN
0% RFNG
MethCluster
Methylation_M
Methylation_H
Methylation_L
Missense_Mutation
Multi_Hit
Nonsense_Mutation
Nonstop_Mutation
Splice_Site
In_Frame_Del
Frame_Shift_Del
Frame_Shift_Ins

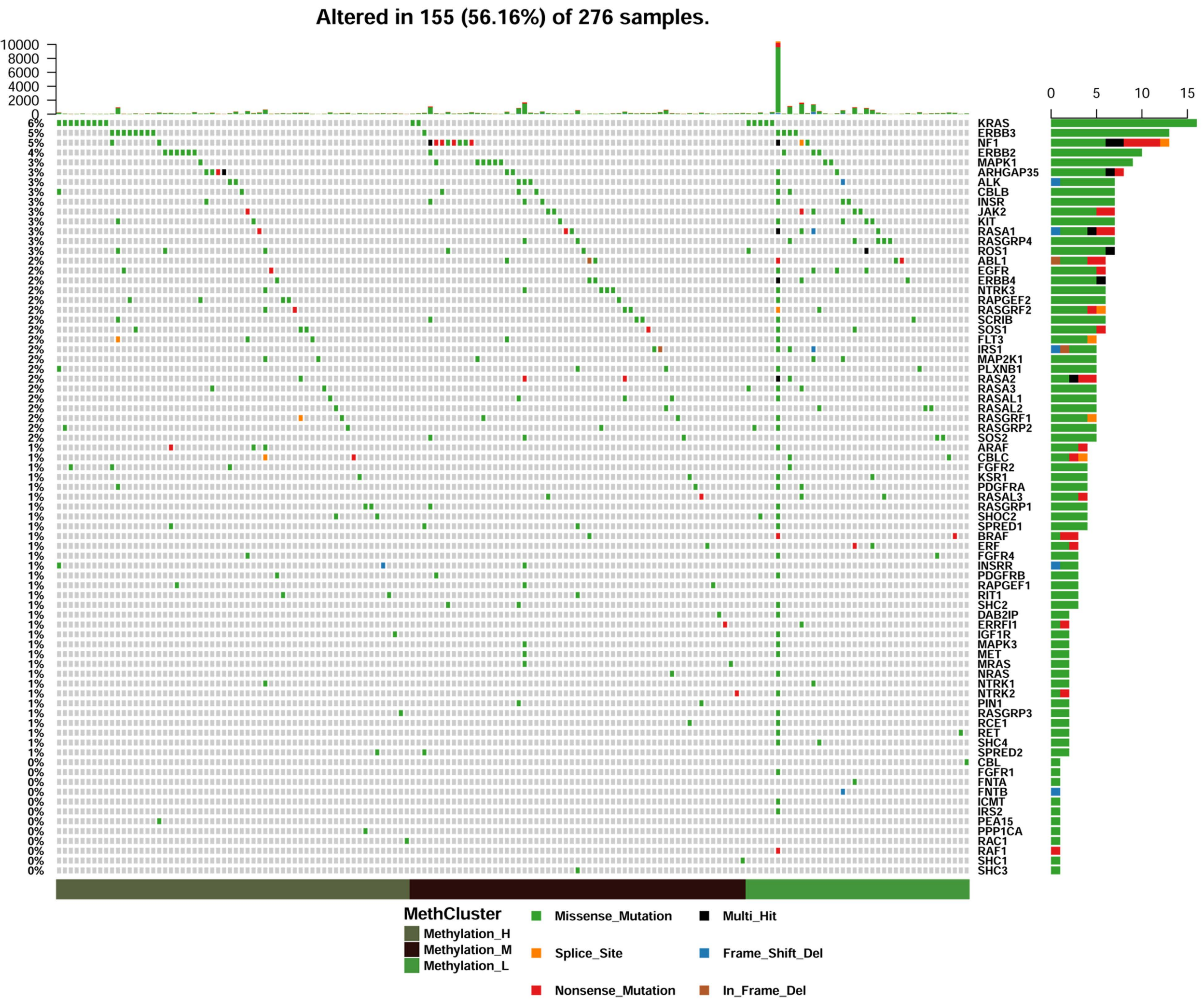

Altered in 155 (56.16%) of 276 samples.
KRAS
ERBB3
NF1
ERBB2
MAPK1
ARHGAP35
ALK
CBLB
INSR
JAK2
KIT
RASA1
RASGRP4
ROS1
ABL1
EGFR
ERBB4
NTRK3
RAPGEF2
RASGRF2
SCRIB
SOS1
FLT3
IRS1
MAP2K1
PLXNB1
RASA2
RASA3
RASAL1
RASAL2
RASGRF1
RASGRP2
SOS2
ARAF
CBLC
FGFR2
KSR1
PDGFRA
RASAL3
RASGRP1
SHOC2
SPRED1
BRAF
ERF
FGFR4
INSRR
PDGFRB
RAPGEF1
RIT1
SHC2
DAB2IP
ERRFI1
IGF1R
MAPK3
MET
MRAS
NRAS
NTRK1
NTRK2
PIN1
RASGRP3
RCE1
RET
SHC4
SPRED2
CBL
FGFR1
FNTA
FNTB
ICMT
IRS2
PEA15
PPP1CA
RAC1
RAF1
SHC1
SHC3
MethCluster
Methylation_H
Methylation_M
Methylation_L
Missense_Mutation
Splice_Site
Nonsense_Mutation
Multi_Hit
Frame_Shift_Del
In_Frame_Del

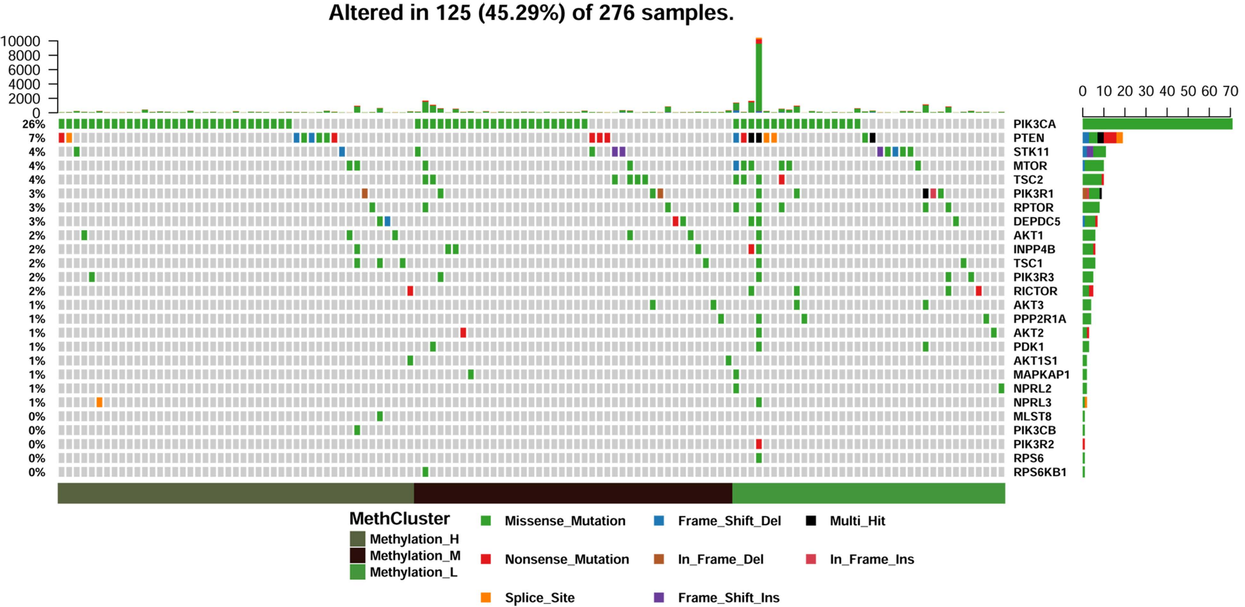

Altered in 125 (45.29%) of 276 samples.
10000
8000
6000
4000
2000
0
0 10 20 30 40 50 60 70
26% PIK3CA
7% PTEN
4% STK11
4% MTOR
4% TSC2
3% PIK3R1
3% RPTOR
3% DEPDC5
2% AKT1
2% INPP4B
2% TSC1
2% PIK3R3
2% RICTOR
1% AKT3
1% PPP2R1A
1% AKT2
1% PDK1
1% AKT1S1
1% MAPKAP1
1% NPRL2
1% NPRL3
0% MLST8
0% PIK3CB
0% PIK3R2
0% RPS6
0% RPS6KB1
MethCluster
Methylation_H
Methylation_M
Methylation_L
Missense_Mutation
Nonsense_Mutation
Splice_Site
Frame_Shift_Del
In_Frame_Del
Frame_Shift_Ins
Multi_Hit
In_Frame_Ins

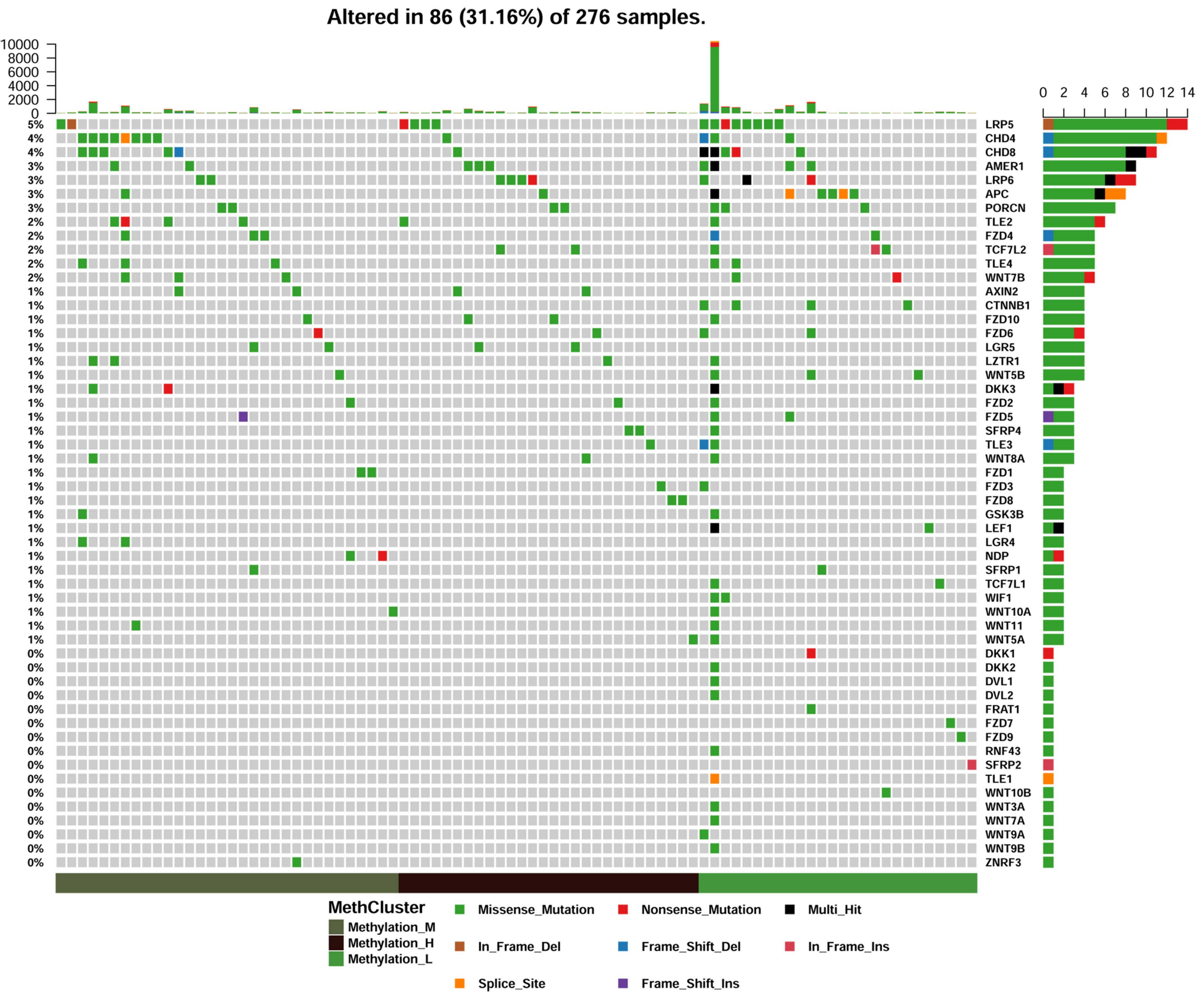
Altered in 86 (31.16%) of 276 samples.
10000
8000
6000
4000
2000
0
0 2 4 6 8 10 12 14
5% LRP5
4% CHD4
4% CHD8
3% AMER1
3% LRP6
3% APC
3% PORCN
2% TLE2
2% FZD4
2% TCF7L2
2% TLE4
2% WNT7B
1% AXIN2
1% CTNNB1
1% FZD10
1% FZD6
1% LGR5
1% LZTR1
1% WNT5B
1% DKK3
1% FZD2
1% FZD5
1% SFRP4
1% TLE3
1% WNT8A
1% FZD1
1% FZD3
1% FZD8
1% GSK3B
1% LEF1
1% LGR4
1% NDP
1% SFRP1
1% TCF7L1
1% WIF1
1% WNT10A
1% WNT11
1% WNT5A
0% DKK1
0% DKK2
0% DVL1
0% DVL2
0% FRAT1
0% FZD7
0% FZD9
0% RNF43
0% SFRP2
0% TLE1
0% WNT10B
0% WNT3A
0% WNT7A
0% WNT9A
0% WNT9B
0% ZNRF3
MethCluster
Methylation_M
Methylation_H
Methylation_L
Missense_Mutation
Nonsense_Mutation
Multi_Hit
In_Frame_Del
Frame_Shift_Del
In_Frame_Ins
Splice_Site
Frame_Shift_Ins

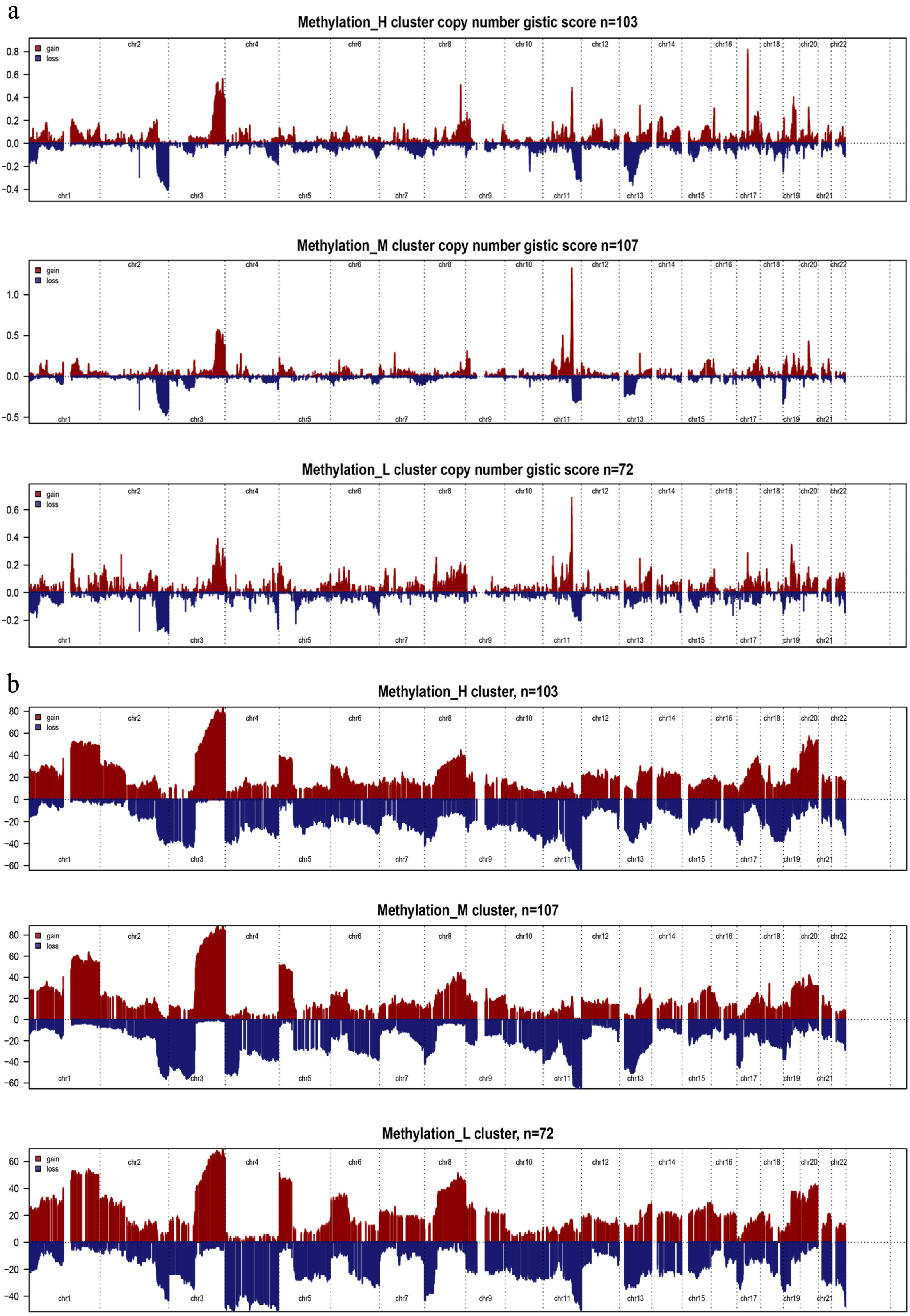
a
Methylation_H cluster copy number gistic score n=103
Methylation_M cluster copy number gistic score n=107
Methylation_L cluster copy number gistic score n=72
gistic score
gain
loss
chr1
chr2
chr3
chr4
chr5
chr6
chr7
chr8
chr9
chr10
chr11
chr12
chr13
chr14
chr15
chr16
chr17
chr18
chr19
chr20
chr21
chr22
b
Methylation_H cluster, n=103
Methylation_M cluster, n=107
Methylation_L cluster, n=72
Frequency

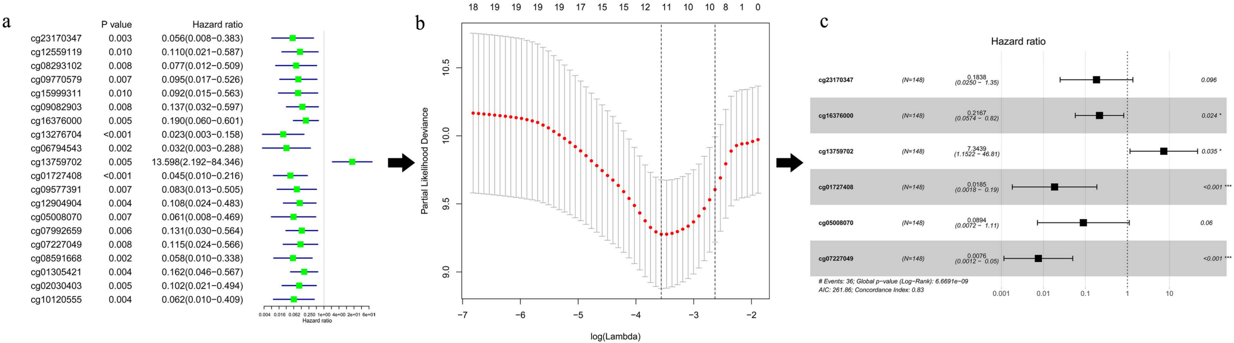
a
P value
Hazard ratio
cg23170347 0.003 0.056(0.008−0.383)
cg12559119 0.010 0.110(0.021−0.587)
cg08293102 0.008 0.077(0.012−0.509)
cg09770579 0.007 0.095(0.017−0.526)
cg15999311 0.010 0.092(0.015−0.563)
cg09082903 0.008 0.137(0.032−0.597)
cg16376000 0.005 0.190(0.060−0.601)
cg13276704 <0.001 0.023(0.003−0.158)
cg06794543 0.002 0.032(0.003−0.288)
cg13759702 0.005 13.598(2.192−84.346)
cg01727408 <0.001 0.045(0.010−0.216)
cg09577391 0.007 0.083(0.013−0.505)
cg12904904 0.004 0.108(0.024−0.483)
cg05008070 0.007 0.061(0.008−0.469)
cg07992659 0.006 0.131(0.030−0.564)
cg07227049 0.008 0.115(0.024−0.566)
cg08591668 0.002 0.058(0.010−0.338)
cg01305421 0.004 0.162(0.046−0.567)
cg02030403 0.005 0.102(0.021−0.494)
cg10120555 0.004 0.062(0.010−0.409)
0.004 0.016 0.062 0.250 1e+00 4e+00 2e+01 6e+01
Hazard ratio
b
18 19 19 19 19 17 15 15 12 11 10 10 8 1 0
Partial Likelihood Deviance
10.5
10.0
9.5
9.0
−7 −6 −5 −4 −3 −2
log(Lambda)
c
Hazard ratio
cg23170347 (N=148) 0.1838 (0.0250 − 1.35) 0.096
cg16376000 (N=148) 0.2167 (0.0574 − 0.82) 0.024 *
cg13759702 (N=148) 7.3439 (1.1522 − 46.81) 0.035 *
cg01727408 (N=148) 0.0185 (0.0018 − 0.19) <0.001 ***
cg05008070 (N=148) 0.0894 (0.0072 − 1.11) 0.06
cg07227049 (N=148) 0.0076 (0.0012 − 0.05) <0.001 ***
Events: 36; Global p-value (Log-Rank): 6.6691e−09
AIC: 261.86; Concordance Index: 0.83
0.001 0.01 0.1 1 10

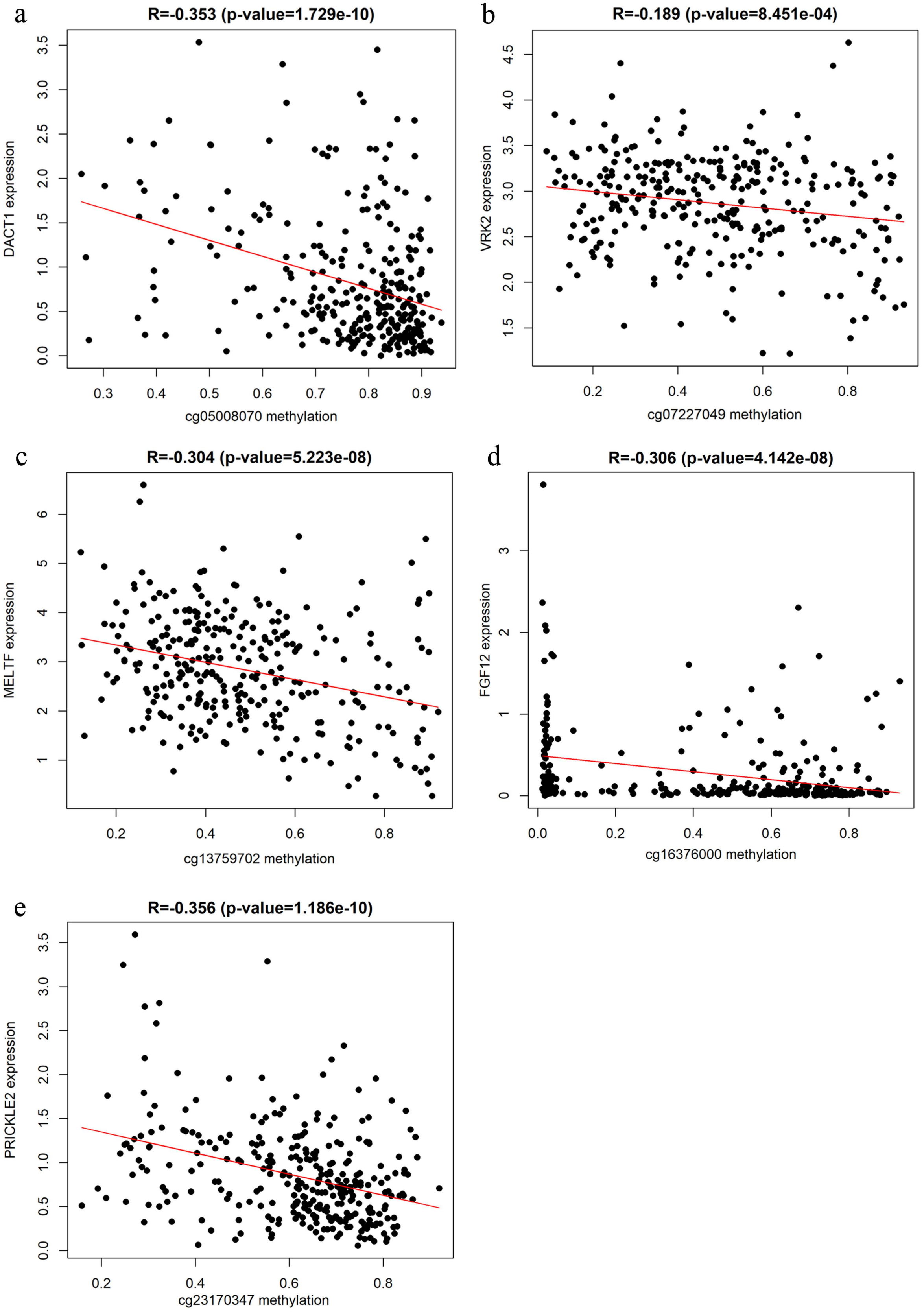
a
R=-0.353 (p-value=1.729e-10)
DACT1 expression
0.0 0.5 1.0 1.5 2.0 2.5 3.0 3.5
0.3 0.4 0.5 0.6 0.7 0.8 0.9
cg05008070 methylation
b
R=-0.189 (p-value=8.451e-04)
VRK2 expression
1.5 2.0 2.5 3.0 3.5 4.0 4.5
0.2 0.4 0.6 0.8
cg07227049 methylation
c
R=-0.304 (p-value=5.223e-08)
MELTF expression
1 2 3 4 5 6
0.2 0.4 0.6 0.8
cg13759702 methylation
d
R=-0.306 (p-value=4.142e-08)
FGF12 expression
0 1 2 3
0.0 0.2 0.4 0.6 0.8
cg16376000 methylation
e
R=-0.356 (p-value=1.186e-10)
PRICKLE2 expression
0.0 0.5 1.0 1.5 2.0 2.5 3.0 3.5
0.2 0.4 0.6 0.8
cg23170347 methylation

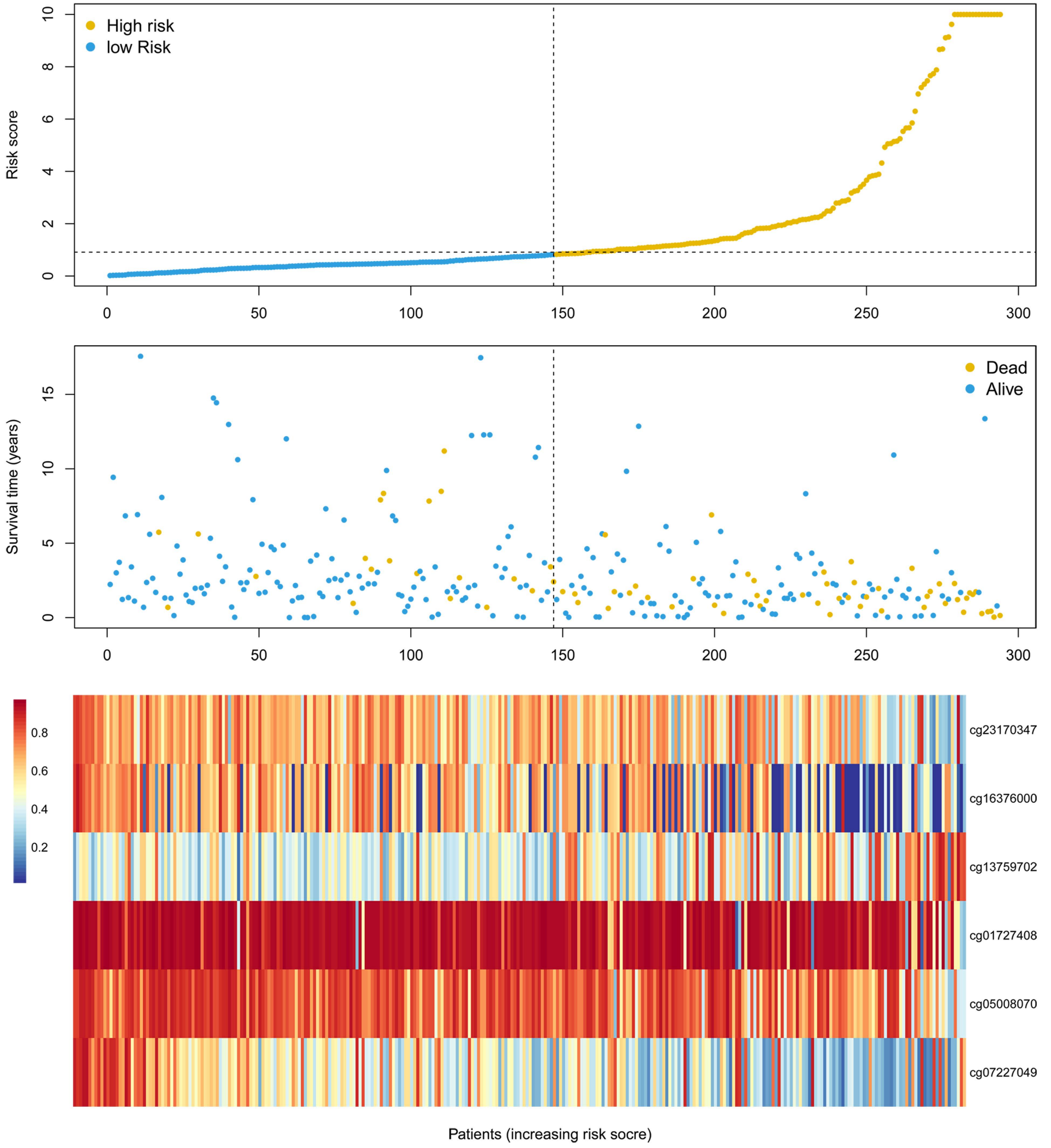

High risk
low Risk
Risk score
Dead
Alive
Survival time (years)
cg23170347
cg16376000
cg13759702
cg01727408
cg05008070
cg07227049
Patients (increasing risk socre)

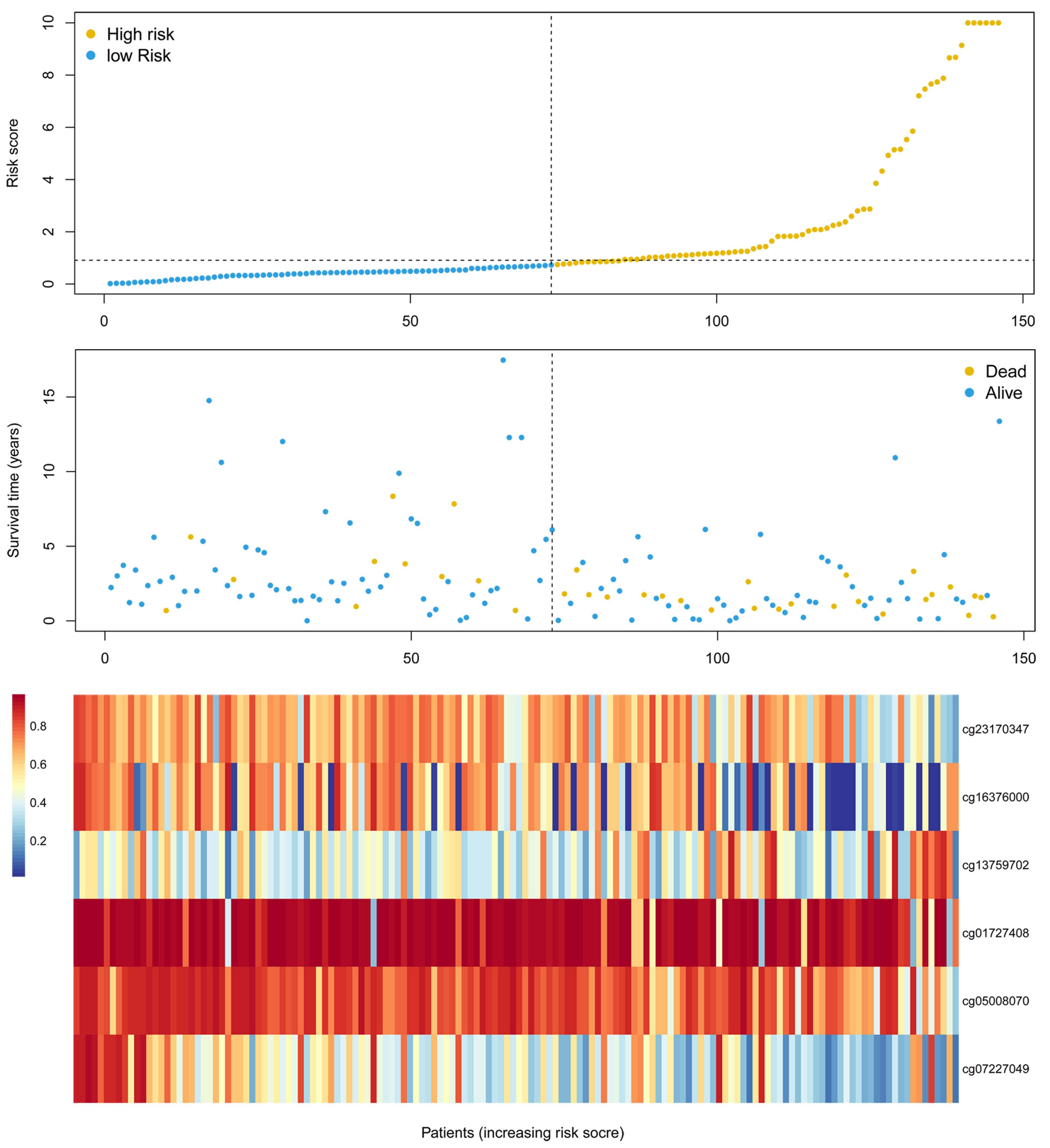

High risk
low Risk
Risk score
0
2
4
6
8
10
0
50
100
150
Dead
Alive
Survival time (years)
0
5
10
15
0.8
0.6
0.4
0.2
cg23170347
cg16376000
cg13759702
cg01727408
cg05008070
cg07227049
Patients (increasing risk socre)

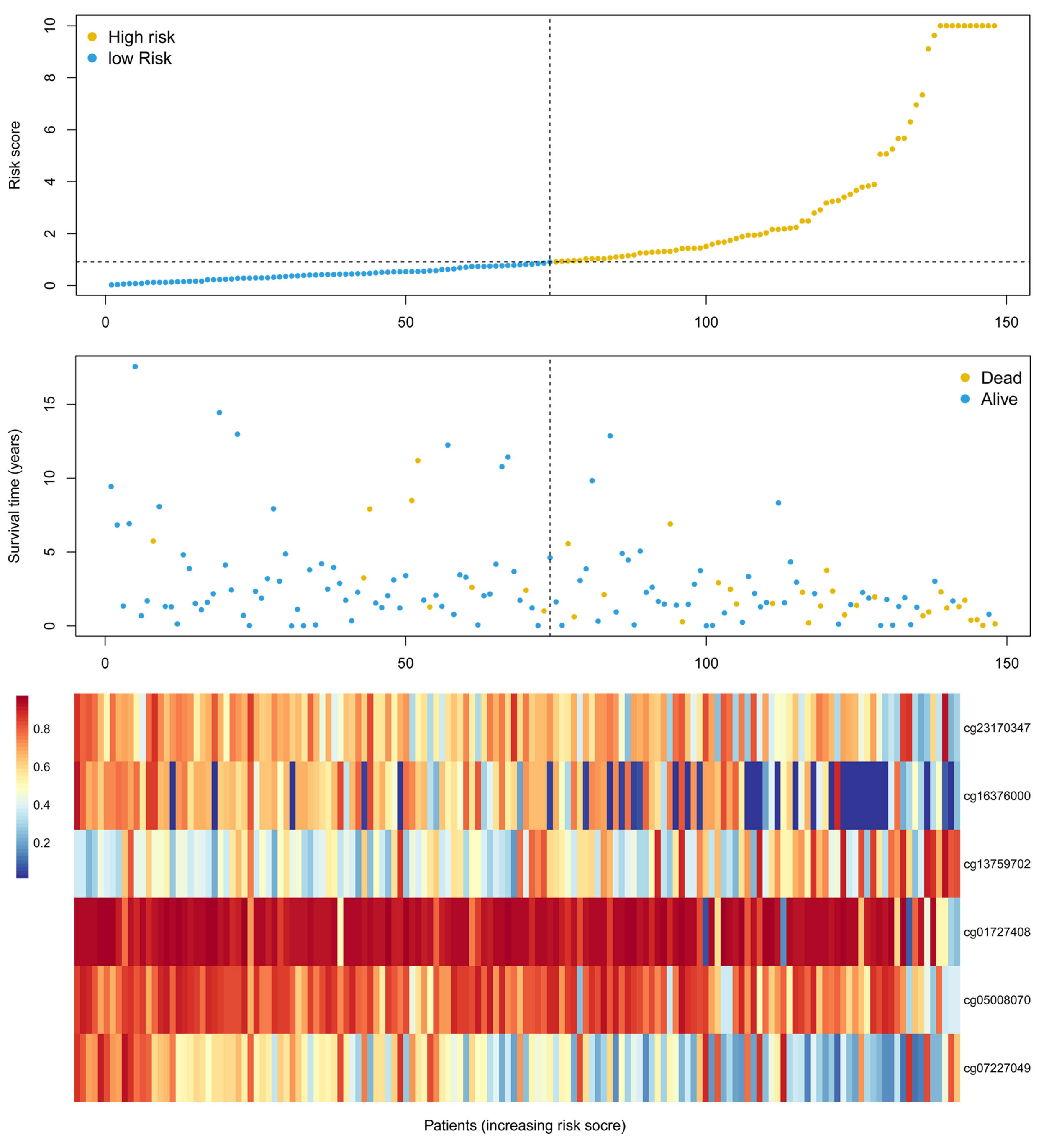

High risk
low Risk
Risk score
0
2
4
6
8
10
0
50
100
150
Dead
Alive
Survival time (years)
0
5
10
15
0
50
100
150
0.2
0.4
0.6
0.8
cg23170347
cg16376000
cg13759702
cg01727408
cg05008070
cg07227049
Patients (increasing risk socre)

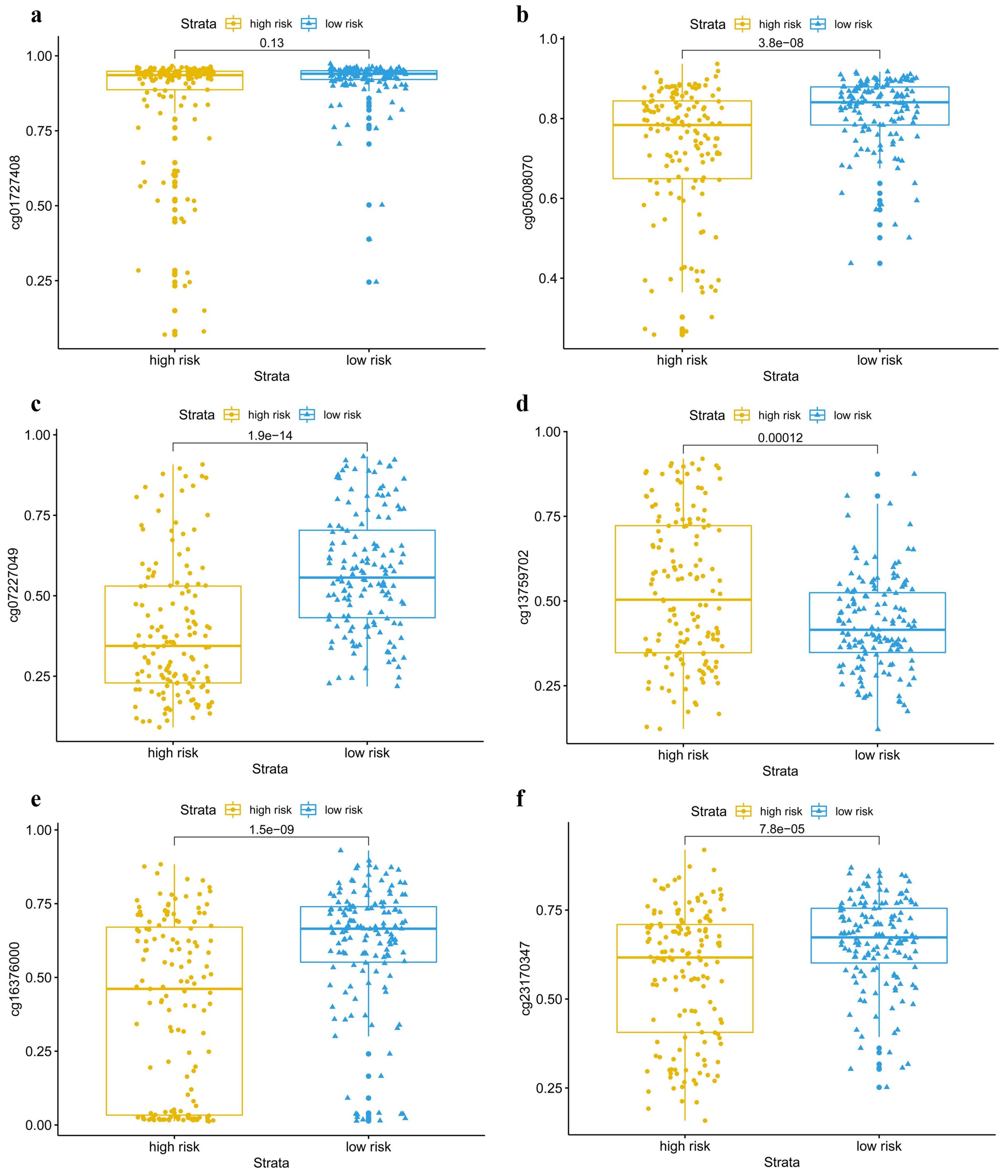
a
Strata high risk low risk
0.13
cg01727408
1.00
0.75
0.50
0.25
high risk
low risk
Strata
b
Strata high risk low risk
3.8e−08
cg05008070
1.0
0.8
0.6
0.4
high risk
low risk
Strata
c
Strata high risk low risk
1.9e−14
cg07227049
1.00
0.75
0.50
0.25
high risk
low risk
Strata
d
Strata high risk low risk
0.00012
cg13759702
1.00
0.75
0.50
0.25
high risk
low risk
Strata
e
Strata high risk low risk
1.5e−09
cg16376000
1.00
0.75
0.50
0.25
0.00
high risk
low risk
Strata
f
Strata high risk low risk
7.8e−05
cg23170347
0.75
0.50
0.25
high risk
low risk
Strata

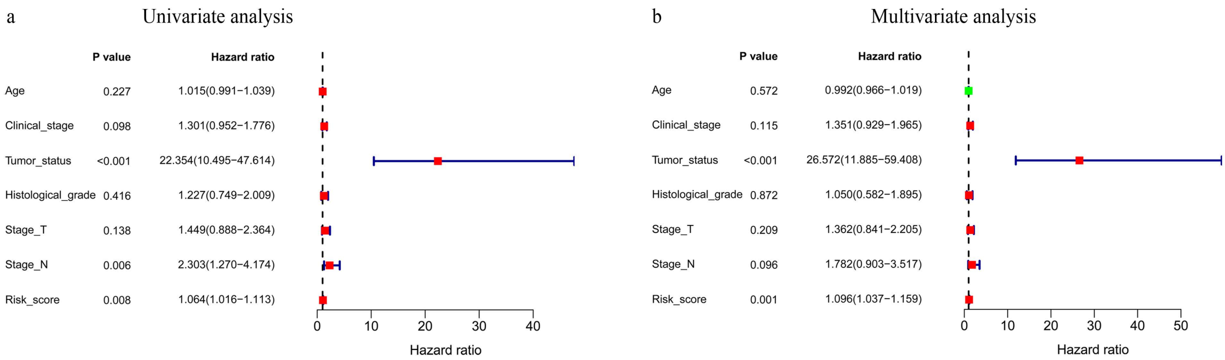

a
Univariate analysis
P value
Hazard ratio
Age 0.227 1.015(0.991−1.039)
Clinical_stage 0.098 1.301(0.952−1.776)
Tumor_status <0.001 22.354(10.495−47.614)
Histological_grade 0.416 1.227(0.749−2.009)
Stage_T 0.138 1.449(0.888−2.364)
Stage_N 0.006 2.303(1.270−4.174)
Risk_score 0.008 1.064(1.016−1.113)
0 10 20 30 40
Hazard ratio
b
Multivariate analysis
P value
Hazard ratio
Age 0.572 0.992(0.966−1.019)
Clinical_stage 0.115 1.351(0.929−1.965)
Tumor_status <0.001 26.572(11.885−59.408)
Histological_grade 0.872 1.050(0.582−1.895)
Stage_T 0.209 1.362(0.841−2.205)
Stage_N 0.096 1.782(0.903−3.517)
Risk_score 0.001 1.096(1.037−1.159)
0 10 20 30 40 50
Hazard ratio

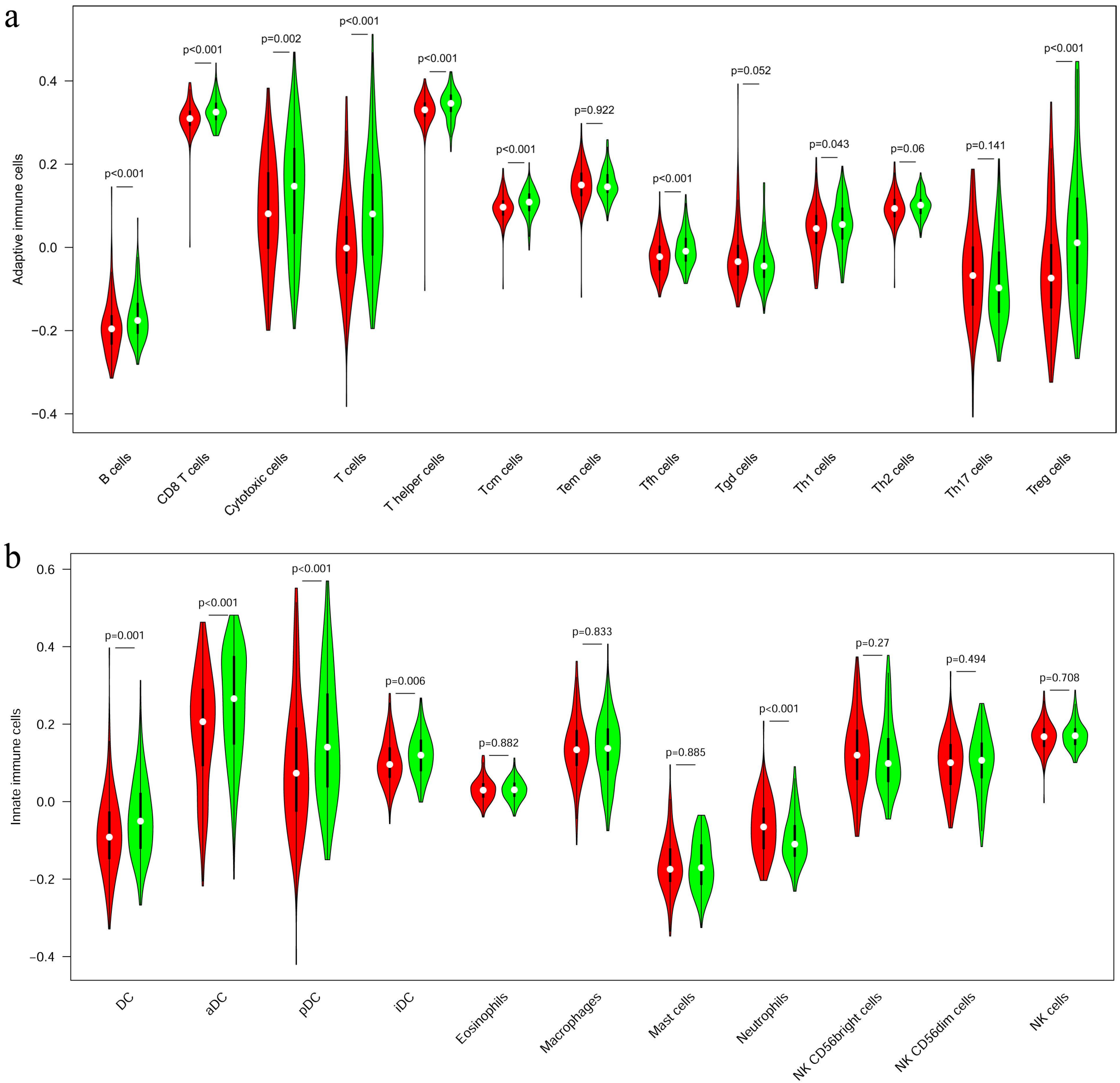
a
Adaptive immune cells
0.4
0.2
0.0
−0.2
−0.4
p<0.001
p<0.001
p=0.002
p<0.001
p<0.001
p<0.001
p=0.922
p<0.001
p=0.052
p=0.043
p=0.06
p=0.141
p<0.001
B cells
CD8 T cells
Cytotoxic cells
T cells
T helper cells
Tcm cells
Tem cells
Tfh cells
Tgd cells
Th1 cells
Th2 cells
Th17 cells
Treg cells
b
Innate immune cells
0.6
0.4
0.2
0.0
−0.2
−0.4
p=0.001
p<0.001
p<0.001
p=0.006
p=0.882
p=0.833
p=0.885
p<0.001
p=0.27
p=0.494
p=0.708
DC
aDC
pDC
iDC
Eosinophils
Macrophages
Mast cells
Neutrophils
NK CD56bright cells
NK CD56dim cells
NK cells

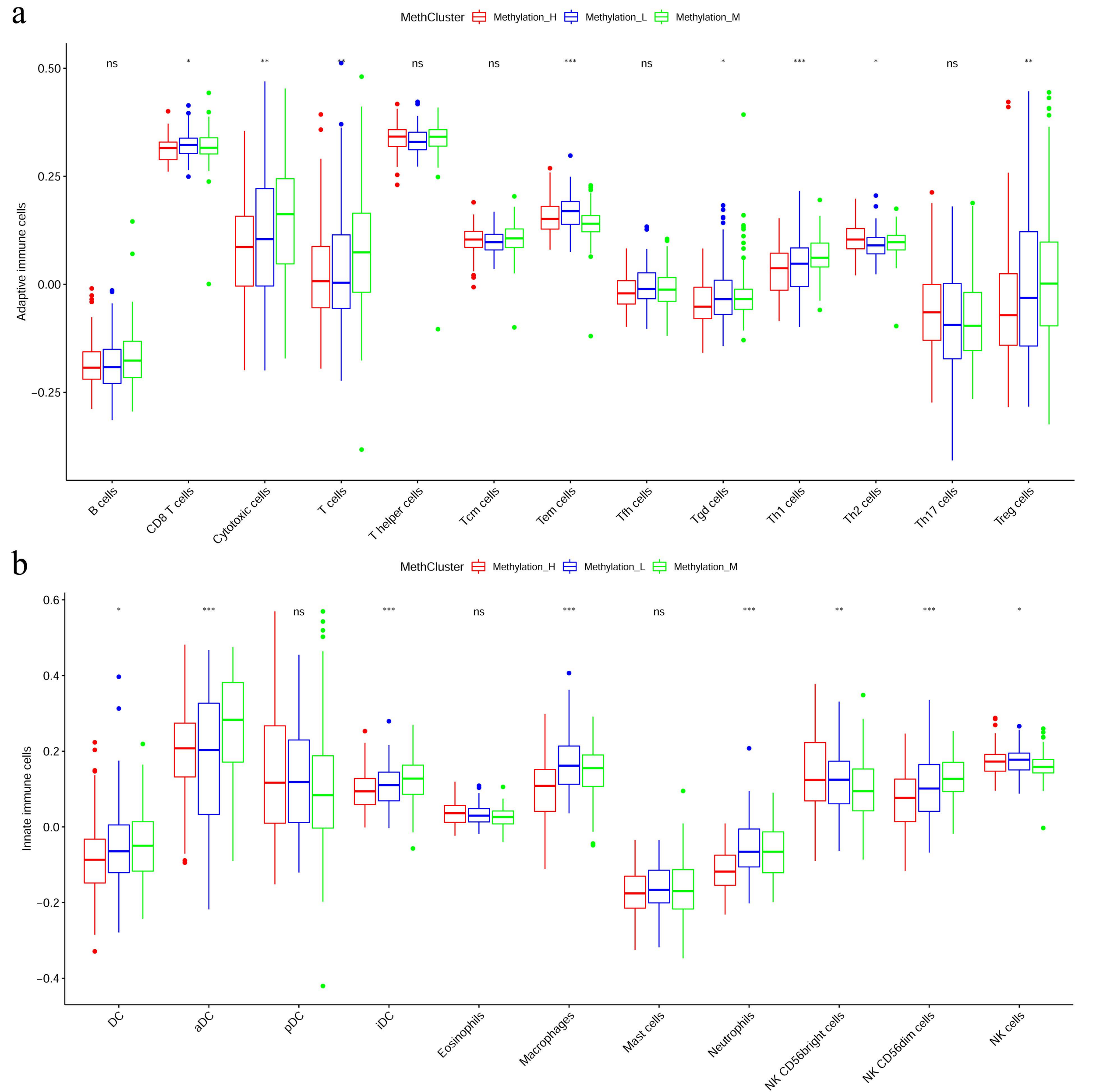
a
MethCluster
Methylation_H
Methylation_L
Methylation_M
Adaptive immune cells
ns
*
**
**
ns
ns
***
ns
*
***
*
ns
**
0.50
0.25
0.00
−0.25
B cells
CD8 T cells
Cytotoxic cells
T cells
T helper cells
Tcm cells
Tem cells
Tfh cells
Tgd cells
Th1 cells
Th2 cells
Th17 cells
Treg cells
b
MethCluster
Methylation_H
Methylation_L
Methylation_M
Innate immune cells
*
***
ns
***
ns
***
ns
***
**
***
*
0.6
0.4
0.2
0.0
−0.2
−0.4
DC
aDC
pDC
iDC
Eosinophils
Macrophages
Mast cells
Neutrophils
NK CD56bright cells
NK CD56dim cells
NK cells

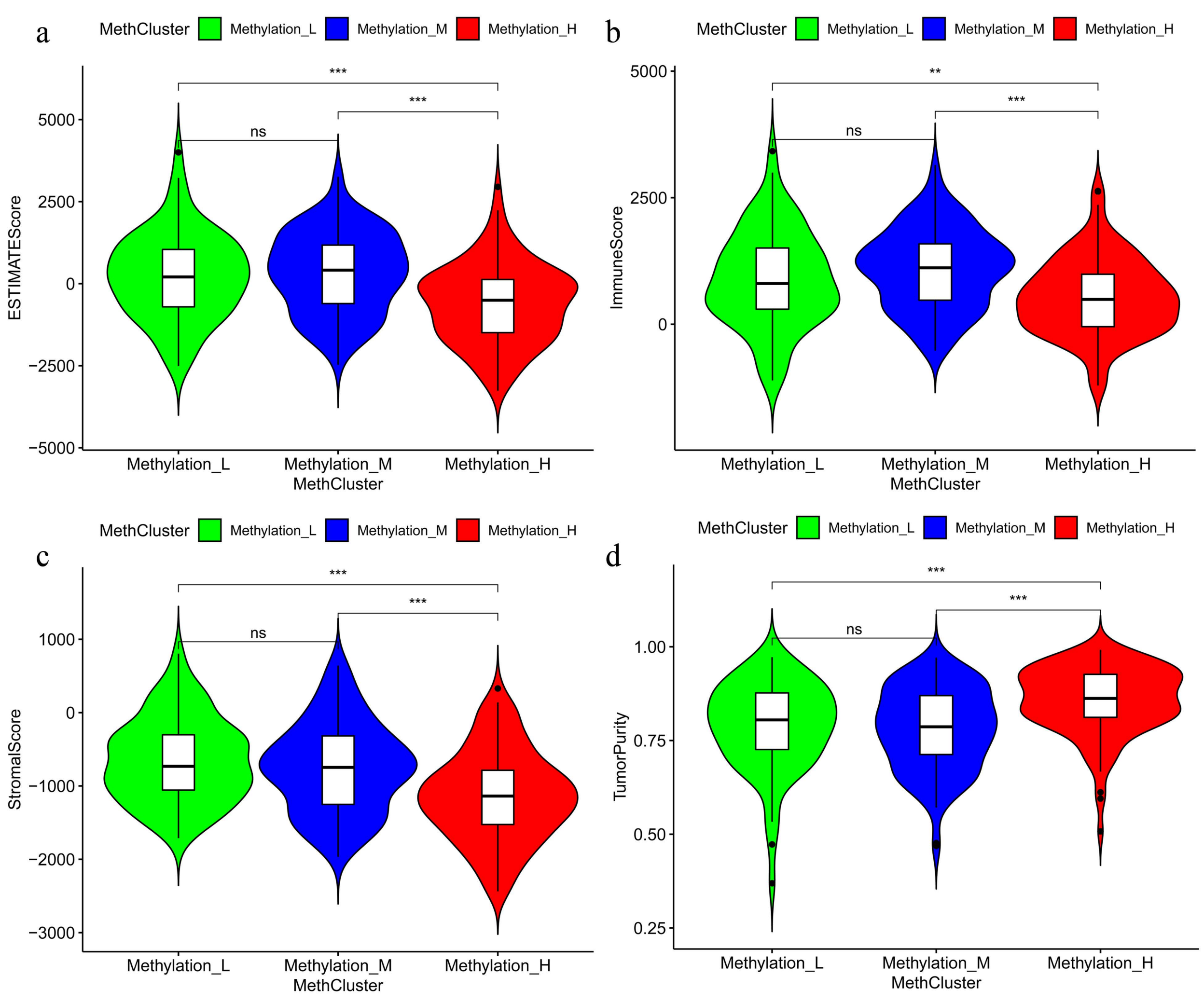
a
MethCluster
Methylation_L
Methylation_M
Methylation_H
ESTIMATEScore
5000
2500
0
−2500
−5000
***
***
ns
Methylation_L
Methylation_M
Methylation_H
MethCluster
b
MethCluster
Methylation_L
Methylation_M
Methylation_H
ImmuneScore
5000
2500
0
**
***
ns
Methylation_L
Methylation_M
Methylation_H
MethCluster
c
MethCluster
Methylation_L
Methylation_M
Methylation_H
StromalScore
1000
0
−1000
−2000
−3000
***
***
ns
Methylation_L
Methylation_M
Methylation_H
MethCluster
d
MethCluster
Methylation_L
Methylation_M
Methylation_H
TumorPurity
1.00
0.75
0.50
0.25
***
***
ns
Methylation_L
Methylation_M
Methylation_H
MethCluster
