# Supplementary material for: HPV‐related methylation‐based reclassification and risk stratification of cervical cancer
Source: Mol Oncol. 2020 Jun 2;14(9):2124–41. doi: 10.1002/1878-0261.12709 (PMC7463306; doi:10.1002/1878-0261.12709)
Supplement: Supplementary file 2 — Table S1. Clinical variables in the training and testing datasets. [file MOL2-14-2124-s002.docx]

**Table S1 Clinical variables in the training and testing datasets.**

| Variables | Group | Entire  (n=294) | Training  (n=148) | Testing  (n=146) | *P* value | Method |
| --- | --- | --- | --- | --- | --- | --- |
| Survival time  (days) |  | 1066 ± 66.90* | 1068 ± 95.54* | 1065 ± 93.97* | 0.98 | *t*-test |
| Vital status | Alive | 223(75.85%) | 112(75.68%) | 111(76.03%) | 0.94 | χ2 test |
|  | Dead | 71(24.15%) | 36(24.32%) | 35(23.97%) |  |  |
| Clinical stage | I | 160(54.42%)) | 79(53.38%) | 81(55.48%) | 0.88 | χ2 test |
|  | II | 65(22.11%) | 35(23.65%) | 30(20.55%) |  |  |
|  | III | 42(14.29%) | 22(14.87%) | 20(13.70%) |  |  |
|  | IV | 21(7.14%) | 10(6.76%) | 11(7.53%) |  |  |
|  | NA | 6(2.04%) | 2(1.35%) | 4(2.74%) |  |  |
| T stage | T1 | 169(57.48%) | 85(57.43%) | 84(57.53%) | 0.88 | χ2 test |
|  | T2 | 77(26.19%) | 41(27.70%) | 36(24.66%) |  |  |
|  | T3 | 17(5.78%) | 9(6.08%) | 8(5.48%) |  |  |
|  | T4 | 10(3.40%) | 4(2.70%) | 6(4.11%) |  |  |
|  | TX | 21(7.14%) | 9(6.08%) | 12(8.22%) |  |  |
| N stage | N0 | 164(55.78%) | 78(52.70%) | 86(58.90%) | 0.49 | χ2 test |
|  | N1 | 64(21.77%) | 33(22.30%) | 31(21.23%) |  |  |
|  | NX | 66(22.45%) | 37(25.00%) | 29(19.86%) |  |  |
| M stage | M0 | 118(40.14%) | 55(37.16%) | 63(43.15%) | 0.19 | χ2 test |
|  | M1 | 10(3.40%) | 3(2.03%) | 7(4.80%) |  |  |
|  | MX | 166(56.46%) | 90(60.81% ) | 76(52.06%) |  |  |
| Age(years) | ≤ 65 | 261(88.78%) | 129(87.16%) | 132(90.41%) | 0.46 | χ2 test |
|  | > 65 | 33(11.22%) | 19(12.84%) | 14(9.59%) |  |  |
| Histological  grade | G1 | 18(6.12%) | 9(6.08%) | 9(6.16%) | 0.48 | χ2 test |
|  | G2 | 130(44.22%) | 65(43.92%) | 65(44.52%) |  |  |
|  | G3 | 118(40.14%) | 62(41.89%) | 56(38.36%) |  |  |
|  | G4 | 1(0.34%) | 0 | 1(0.69%) |  |  |
|  | GX | 27(9.18%) | 12(8.11%) | 15(10.27%) |  |  |
| Histological  type | Squamous cell carcinoma | 243(82.65%) | 121(81.76%) | 122(83.56%) | 0.91 | χ2 test |
|  | Adenosquamous | 6(2.04%) | 3(2.03%) | 3(2.06%) |  |  |
|  | Adenocarcinoma | 45(15.31%) | 24(16.22%) | 21(14.38%) |  |  |
| HPV status | Positive | 167(56.80%) | 85(57.43%) | 82(56.16%) | 0.56 | χ2 test |
|  | Negative | 9(3.06%) | 6(4.05%) | 3(2.06%) |  |  |
|  | NA | 118(40.14%) | 57(38.51%) | 61(41.78%) |  |  |
| Tumor status | Tumor free | 199(67.69%) | 99(66.89%) | 100(68.49%) | 0.11 | χ2 test |
|  | With tumor | 80(27.21%) | 45(30.41%) | 35(23.97%) |  |  |
|  | NA | 15(5.10%) | 4(2.70%) | 11(7.53%) |  |  |

**NA:** Not Available**; TX:** unknown T stage**; MX:** unknown M stage**, GX:** unknown histological grade**; NX:** unknown N stage. * The data are presented as mean ± SE
